# Fibroblast Growth Factor 19 Disrupts Cartilage Development Via the FGFR4/β-catenin Axis

**DOI:** 10.7150/ijbs.110133

**Published:** 2025-07-04

**Authors:** Hao Chen, Yujia Cui, Jiazhou Li, Mengmeng Duan, Caixia Pi, Xuedong Zhou, Jing Xie

**Affiliations:** State Key Laboratory of Oral Diseases, West China Hospital of Stomatology, Sichuan University, Chengdu 610041, China.

**Keywords:** fibroblast growth factor 19, growth plate, chondrogenesis, Wnt/β-Catenin

## Abstract

Fibroblast growth factor 19 (FGF19) has received increasing attention in metabolic disorders of the skeletal system, but its role in cartilage development is poorly understood. In the present study, we used *ex vivo* metatarsal organ model for nascent cartilage and an AAV-FGF19 overexpression model for adolescent growth plates to demonstrate the influence of FGF19 on cartilage development. We found that FGF19 could impair chondrocyte maturation at the neonatal stage and decrease growth plate thickness at the adolescent stage. FGF19 reduces chondrogenic differentiation of mesenchymal stem cells and the chondrocyte maturation via downregulation of Wnt/β-catenin signalling. FGF19-mediated chondrocyte maturation and cartilage differentiation require the participation of FGFR4 with the aid of β-klotho (KLB). FGF19 signalling entered the cytoplasm through FGFR4, activated the expression of SFRP1, WIF1 and DKK2, which are antagonists of β-catenin signalling, and hindered chondrocyte proliferation and cartilage growth. This study demonstrates for the first time that FGF19 inhibits cartilage development through the FGFR4/β-catenin axis, providing evidence for the vital role of FGF19 in growth plate chondrogenesis and endochondral ossification.

## Introduction

Endochondral ossification is fundamental to the formation of long bones. During early embryonic development, the prototype of long bones is formed by aggregates of mesenchymal cells, which differentiate into chondrocytes and generate cartilage matrix, forming a cartilage anlage 1. In the neonatal stage, most long bones are still composed of cartilage. With the emergence of the primary ossification center, growth plates develop at both epiphyseal ends, marking the onset of endochondral bone formation 2. Chondrocytes in the growth plate are arranged in different zones, namely, the resting zone, proliferative zone and hypertrophic zone, according to different stages of differentiation 2. During adolescence, the bone grows rapidly and the growth plate is the main growth area where cartilage continues to proliferate and ossify until the growth plate closes completely in adulthood, when bone growth ultimately stops 3. This process is critical for the formation of bone length and shape.

A variety of signaling pathways, such as Indian hedgehog (IHH), fibroblast growth factor (FGF), parathyroid hormone-related protein (PTHrP), bone morphogenetic proteins (BMPs), and transforming growth factor-β (TGF-β), coordinate to shape the early cartilage anlage and regulate the development and maturation of the growth plate 4-6. Disruption in this intricate network can result in skeletal abnormalities and defects in cartilage or bone formation. The key transcription factor during the cartilage formation phase is SOX9 7. It works together with SOX5 and SOX6 (termed the Sox trio), dominates the differentiation of mesenchymal cells to chondrocytes and initiates the transcription of collagen genes (e.g., *Col2a1*), which drive the formation of the cartilage matrix 8. TGF-β stabilises Sox9 in chondrocytes by activating the classical Smad and nonclassical p38 pathways 9. Moreover, BMP is required for cartilage formation and maintenance of SOX9 expression 6. With the formation of the primary ossification centre, the signalling molecule IHH and its regulator PTHrP subsequently become vital regulators 4. IHH activates the GLI transcription factor family by binding to its receptor patched 1 (PTCH1), driving the transformation of chondrocytes into osteoblasts and promoting the ossification of the cartilage matrix 10. PTHrP forms a negative feedback regulatory loop with IHH to maintain the balance between the preosteogenic and proliferative zones of the growth plate and maintain the normal proliferation and differentiation status of chondrocytes 4,11. BMP enhances IHH signaling through this feedback loop and simultaneously suppresses FGF signaling, which inhibits chondrocyte proliferation and hypertrophic differentiation 6.

Among the multiple regulatory pathways involved in endochondral ossification, FGF signalling has received increasing attention due to the spatiotemporal roles of its tyrosine kinase receptors (FGFR1-4) during distinct stages of chondrogenesis 12. During early cartilage development, FGFR1 and FGFR2 are highly expressed in condensed mesenchyme, whereas FGFR3 and FGFR4 are not yet detectable at this stage 13. FGFR1 plays a critical role in promoting mesenchymal condensation and initiating hypertrophic differentiation, as evidenced by impaired hypertrophy in FGFR1 conditional knockout mice at this stage 14. FGFR2 is essential for limb bud initiation, and its conditional inactivation in limb mesenchyme leads to reduced postnatal bone growth. However, because chondrocyte proliferation and proliferative zone length are not significantly affected, FGFR2 has a redundant function in resting or proliferating chondrocytes after birth 15. As mesenchymal cells differentiate into chondrocytes, FGFR3 expression is activated alongside chondrogenic markers such as SOX9 and COL2A1, while FGFR2 expression diminishes 16. In the organized growth plate, FGFR3 is highly expressed in the proliferative and prehypertrophic zones and exerts a dual role by promoting chondrocyte proliferation and hypertrophy in the early stage of growth plate establishment 17, but subsequently suppresses both proliferation and hypertrophic differentiation after the formation of the secondary ossification centre 18. These stage-specific functions of FGFR3 are mirrored by its ligands FGF9 and FGF18. Early in development, FGF9-/- and FGF18-/- mice exhibit impaired chondrocyte proliferation and hypertrophy 19,20, whereas at later stages, the hypertrophic zone is expanded in these mutants, reflecting the inhibitory role of FGFR3 signaling in late-stage cartilage development 21. Compared to the well-characterized roles of FGFR1-3, studies focusing on FGFR4 in cartilage development are relatively limited. Current evidence indicates that FGFR4 is primarily expressed in the resting and proliferative zones of the growth plate. It has been reported that FGF18 may regulate developmental processes by modulating chondrocyte autophagy through FGFR4^22^. In our previous study, we demonstrated that FGF19 requires FGFR4 to regulate the chondrocyte cell cycle 23. However, the specific role of individual endocrine FGF members, such as FGF19, in cartilage development is still largely unknown.

FGF19 has a weak affinity for heparin and is thus able to enter the systemic circulation to exert hormone-like effects in the body 24. Due to its limited receptor-binding capacity, FGF19 requires the co-receptor β-Klotho (KLB) to enhance its interaction with target FGFRs 25. FGF19 primarily acts on the organs where KLB and FGFR4 are coexpressed 26,27. FGF19 was firstly discovered in the human foetal brain 28, and subsequent studies confirmed its presence in several fetal tissues, including skin, retina, small intestine, placenta, umbilical cord, and cartilage 29. Notably, FGF19 expression has been detected in fetal limb cartilage and growth plates, indicating a potential role in skeletal development 30. Erika Yeh et al. reported that the unnatural binding of FGF19 to FGFR2 affects the osteogenic differentiation of cells by influencing the expression of RUNX2 and BMP inhibitors 31. More recently, Guo A et al. demonstrated that FGF19 enhances osteogenesis by activating the Wnt/β-catenin signaling pathway, helping to counteract bone loss associated with ageing and obesity 32. Our previous study revealed that FGF19 inhibits chondrocyte proliferation, mediates the arrest of the chondrocyte cell cycle at the G2 phase, and affects chondrocyte metabolism by enhancing chondrocyte mitochondrial biosynthesis and fusion 23, 33.

Although FGF19 has been implicated in cartilage development and bone metabolism, its precise role in cartilage development, particularly in the growth plate development of neonates and adolescents, remains poorly understood. There is currently a lack of direct evidence to elucidate the regulatory mechanism of FGF19 in cartilage development. This knowledge gap limits our understanding to utilize FGF19 as therapeutic interventions targeting skeletal disorders. In the present study, we aimed to investigate the role of FGF19 in the development of growth plates during the neonatal and adolescent stages and elucidate the underlying biomechanism involved. This study enhances the understanding of the role of FGF family members in skeletal development and provides some clues for treatments for cartilage diseases.

## Materials and Methods

### Metatarsal bone extract and culture

The study was approved by The Sichuan University Institutional Review Board (IRB at the West China Hospital of Stomatology, No. WCHSIRB-OT-2020-048). Metatarsal bones (2nd, 3rd, and 4th) were isolated from neonatal (P0) C57/BL mice under a dissecting microscope. The bones were randomly assigned into experimental groups (5-10 bones per group) and cultured in 24-well plates with 0.5 ml of α-MEM (SH30070, HyClone, UT, USA), supplemented with 0.05 mg/ml ascorbic acid (1043003, Merck, Darmstadt, Germany), 1 mM sodium glycerophosphate (G9422, Merck, Darmstadt, Germany), 0.2% bovine serum albumin, and 1% penicillin-streptomycin solution (SV30010, HyClone, UT, USA). The culture medium was refreshed every other day. Metatarsal bones were cultured in the absence or presence of 200ng/ml KLB (2619-KB, Bio-Techne, Shanghai, China) and FGF19 (#100-32, Pepro-tech, NJ, USA), with or without BLU9931 (5 μM, 5387760001, Sigma, MO, USA). BLU9931 was pre-incubated for 30 minutes before FGF19 and KLB addition. The total culture duration was 7 days. The concentrations of FGF19 and β-Klotho (both at 200 ng/ml) were determined based on our previous experiments, in which a detailed dose-response analysis was conducted to identify the optimal dosage 23. This concentration reliably induced cellular responses without causing non-specific toxicity. For β-Klotho, we followed previously reported protocols and used it at the same concentration as FGF19^34^. These concentrations were applied uniformly across all *in vitro* and *ex vivo* experiments to ensure consistency and comparability.

### Histological analyses

In order to accurately calculate the metatarsal growth rate, we captured images of the metatarsal bones at the beginning and end of the 7-day period using an Olympus SZX16 stereomicroscope (SZX16, Olympus, Tokyo, Japan) with 2× magnification. Bone length was assessed using ImageJ software. For histological analysis, H&E staining was performed using hematoxylin (No. 03971, Sigma, St. Louis, MO, and eosin (HT110232, Sigma, St. Louis, MO). Safranin O staining was applied to visualize cartilage proteoglycans. Prior to this, sections were stained with Weigert's iron hematoxylin (HT1079, Sigma), followed by washing with 1% acid-alcohol. Sections were then stained with 2% Safranin O for 30 minutes, and counterstained with 0.02% fast green. The delineation of metatarsal growth plate layers was based on cellular morphological differences as previously described 35-37. Heights of the resting zone (RZ), proliferative zone (PZ), and hypertrophic zone (HZ) were measured. Quantitative analysis was conducted by a single blinded observer.

### Immunofluorescence analysis

Immunofluorescence was performed as described previously 38 to analyze protein localization in joint tissues. Tissue sections underwent antigen retrieval at 100°C for 10 minutes, permeabilization with 5% Triton X-100 for 5 minutes, and blocking with serum after H2O2 (0.5%) incubation. Primary antibodies were incubated either for 2 hours at 37°C or overnight at 4°C. The primary antibodies included FGF19 (1:200, ab225942, Abcam, Cambridge, UK), KLB (1:200, SAB2108630, Sigma, MO, USA), Ki67 (1:200, AF1738, Beyotime, Shanghai, China), PCNA (1:200, 200947-2E1, ZenBio, Chengdu, China), Collagen type II (1:200, SAB4500366, Sigma, MO, USA), SOX9 (1:200, ab185966, Abcam, Cambridge, UK), Collagen type X (1:200, ab182563, Abcam, Cambridge, UK), MMP13 (1:200, ab39012, Abcam, Cambridge, UK), ALP (1:200, ab224335, Abcam, Cambridge, UK) and FGFR4 (1:200, 381880, Zenbio, Chengdu, China). The secondary antibodies were anti-rabbit IgG H&L (Alexa Fluor® 488, ab150073, and Alexa Fluor® 647, ab150075, 1:200 dilution). Nuclear staining was performed using 4',6-Diamidino-2-phenylindole (DAPI, D9542, Sigma, MO, USA). Fluorescent staining was then observed using confocal laser scanning microscopy (CLSM; FV3000, Olympus, Japan).

For the cells, β-catenin (1:200, ab32572, Abcam, Cambridge, UK) antibodies were incubated overnight at 4°C (1:200 dilution). After blocking the cells with 5% bovine serum albumin for an hour, the cells were then incubated with Alexa Fluor 647-conjugated anti-rabbit fluorescent secondary antibody (1:200, ab150075, Abcam, Cambridge, UK) on the next day for 2 hours at room temperature. Phalloidin (FITC, 1:40, A12379, Thermo, USA) and Dapi were used to stain the cytoskeleton and the nuclei. The stained cells were then imaged using CLSM.

### AAV- FGF19 animal model

FGF19-overexpressing AAV vectors were generated by GENECHEM (Shanghai, China). AAV injection was achieved as previously described 39. AAV-FGF19 (1 × 10^11^ v.g. per mL, 5 μl per joint) was injected intra-articularly into 4-week-old mice. Knee joints were harvested at 8 weeks for analysis.

### Micro-CT (μ-CT) bone analysis

Joints were fixed in 4% paraformaldehyde (PFA) overnight, washed, dehydrated with ethanol, and scanned. Imaging was performed using X-ray micro-CT under the following settings: 55 kVp tube voltage, 145 μA current, 220 mg·cm^-3^ threshold, 200 ms integration time, Gaussian filter σ=0.8, support=1.0.

### Cell isolation and chondrogenic induction

We obtain BMSCs as previously described 40. In brief, bone marrow cavities were rinsed with Dulbecco's modified Eagle medium (DMEM; SH30070, HyClone, UT, USA) after quickly removing both femur and tibia from 3-week-old male C57/BL mice under sterile conditions. The collected bone marrow cell suspension was centrifuged, filtered through a 200-mesh filter, and inoculated in a culture flask, which was placed in a 37 °C, 5% CO_2_ environment. BMSCs from passage 3 to 5 were selected for subsequent experiments. To evaluate the chondrogenesis ability, BMSCs were cultured in chondrogenic medium (CM, MUXMX-9004, Cyagen Biosciences, CA) following the manufacturer's instructions.

Neonatal chondrocytes were isolated from knee cartilage of 0-3 day-old mice as previously described 41,42. Tissues were enzymatically digested with 0.25% trypsin for 30 minutes and 0.2% type II collagenase overnight at 37°C. The cells were cultured in DMEM with 10% FBS, and only passage 1 and 2 cells were used for experiments.

### RNA Sequencing

For RNA isolation, cells treated with FGF19 and KLB were lysed in TRIzol (15596018, Thermo, MA, USA). RNA integrity was checked using the RNA Nano 6000 kit and Bioanalyzer 2100 system. Samples were submitted to Shanghai Lifegenes Biotechnology for transcriptomic analysis. Differentially expressed genes were analyzed using GO and KEGG enrichment through the DAVID database (p<0.05, |FoldChange| > 1.5).

### Alcian blue assay and safranin O staining

To assess the production level of cartilage-specific proteoglycans, we conducted Alcian blue and Safranin O staining. For the Alcian blue assay, the BMSCs were first rinsed with PBS three times, fixed with 4% paraformaldehyde for 30 minutes, and then equilibrated with 3% glacial acetic acid for another 30 minutes. The Alcian blue solution was then added for overnight incubation, followed by rinsing with 3% glacial acetic acid three times. Another measure of chondrogenic differentiation, Safranin O staining, was also performed. For this assay, BMSCs were differentiated in 1% acetic acid for 5 minutes and then stained with 1% Safranin O solution (HT90432, Sigma-Aldrich, MO) for 30 minutes. The cell samples were finally washed with 95% ethanol. We observed and captured representative images of Alcian blue and Safranin O staining using an Olympus microscope (Olympus IX71, Japan).

### Western blot analysis

BMSCs were treated with 200 ng/ml of FGF19 and KLB. Pre-treatment with 5 μM BLU9931 (5387760001, Sigma, MO, USA) or 20 mM LiCl (L9650, Sigma, MO, USA) was performed where necessary. Proteins were extracted using RIPA lysis buffer (68117726, Biosharp, Hefei, China). Concentrations were determined by BCA assay, and equal amounts (20 ng) of protein were loaded onto 10% SDS-PAGE gels. Proteins were transferred to PVDF membranes, blocked with 5% skim milk for 2 hours, and probed overnight at 4°C with the following primary antibodies: rabbit anti-Collagen type II (1:800, SAB4500366, Sigma, MO, USA), rabbit anti-SOX9 (1:1000, ab3697, Abcam, Cambridge, UK), rabbit anti-SFRP1 (1:1000, ab267466, Abcam, Cambridge, UK), rabbit anti-WIF1 (1:1000, 823575, Zenbio, Chengdu, China), rabbit anti-DKK2 (1:1000, HPA052611, Sigma, MO, USA), Rabbit anti-β-catenin (1:1000, ab32572, Abcam, Cambridge, UK), and Mouse anti-β-actin (1:1000, sc-47778, Santa Cruz, USA). After four rinses with TBST (0.1% Tween-20/TBS), the PVDF membrane was placed in the anti-IgG-HRP and incubated for 2 h. The blots' signals were obtained using Super signal reagent (32106, Pierce, IL, USA), and Image-J software was used for quantitative analysis.

### Statistical analysis

GraphPad Prism 8.0 and SPSS 22.0 (Statistical Package for the Social Sciences) were used for statistical analysis in this study. The study's data is presented as the mean ± SD. All statistical analyses were based on two-tailed Student's t-tests. The statistical threshold was set as 0.05 for each analysis.

## Results

### FGF19 impairs chondrocyte maturation and nascent bone development

**Figure 1:**
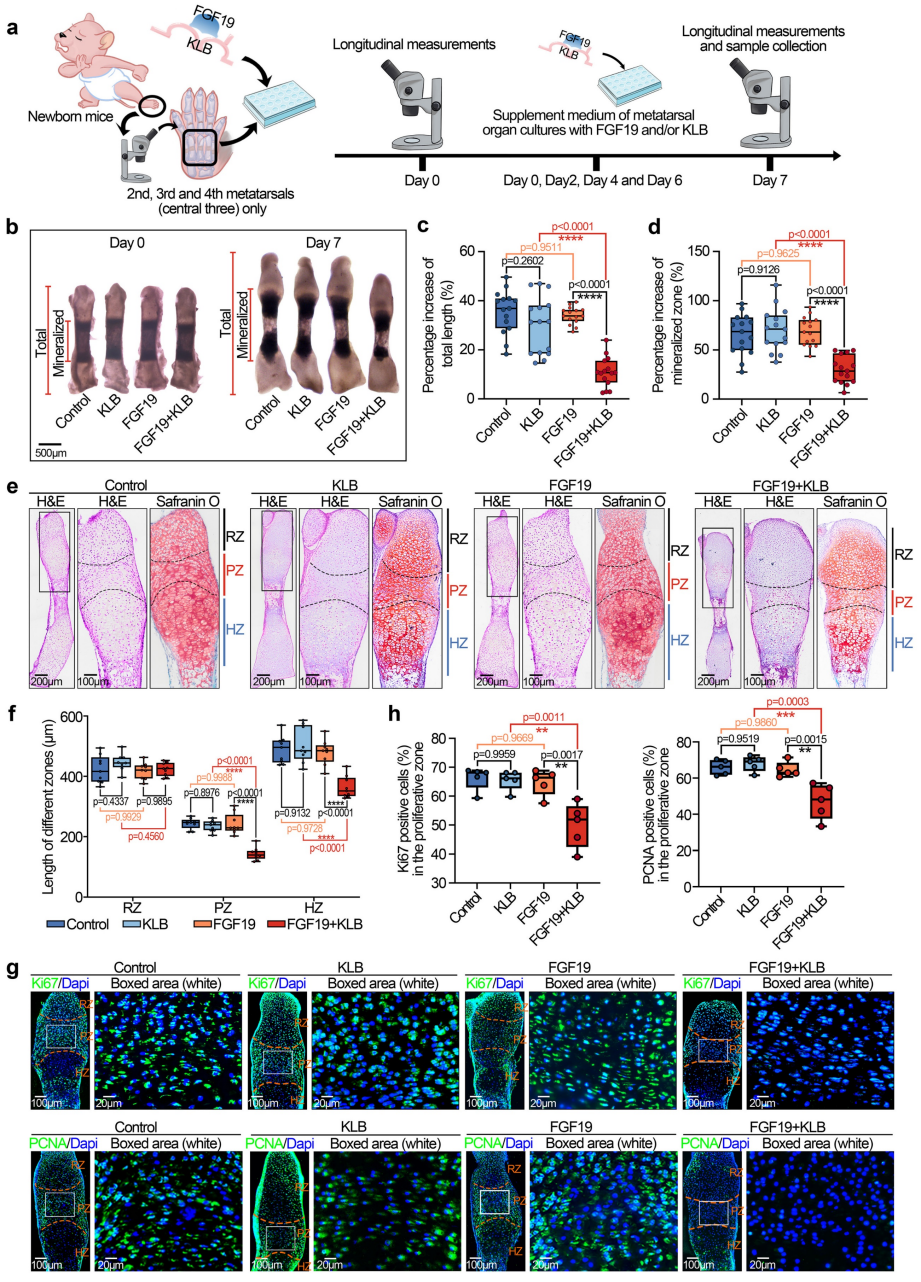
FGF19 reduces metatarsal longitudinal growth and growth plate chondrocyte proliferation *ex vivo*.** a.** Schematic diagram showing the model of *ex vivo* metatarsal organ culture induced by FGF19 and the detailed sample collection procedure. **b.** Representative stereomicroscope images showing the shortened length of metatarsal bone induced by FGF19 (200 ng/ml) for 7 days with KLB (200 ng/ml). **c-d.** Quantitative analysis of the overall growth of metatarsal bones (bone total length in (c) and mineralized region in (d)) induced by FGF19 (200 ng/ml) for 7 days with KLB (200 ng/ml). **e.** H&E and Safranin O staining showing the length changes in the resting zone (RZ), proliferative zone (PZ) and hypertrophic zone (HZ) of metatarsal bones induced by FGF19 (200 ng/ml) for 7 days with KLB (200 ng/ml). **f.** Quantitative analysis of the lengths of the RZ, PZ and HZ of the metatarsals. **g.** Immunofluorescence showing the level and distribution of Ki67 and PCNA in the metatarsal growth plate induced by FGF19 (200 ng/ml) for 7 days with KLB (200 ng/ml). **h.** Quantitative analysis of the percentage of Ki67- and PCNA-positive cells in metatarsal growth plates. The data in **c, d, f** and **h** are presented as box (from 25, 50 to 75%) and whisker (standard deviation, SD). The significance analysis in **c, d, f** and **h** was based on two-tailed Student's t tests. All results in **b, e** and **g** were obtained from at least five independent experiments (n ≥ 5).

Given the advantages of metatarsal organ culture, which facilitates the observation of the pronounced postnatal growth of bone influenced by local biofactors 43, we applied this method to investigate the direct effect of FGF19 on postnatal bone development. We first isolated neonatal mouse (0-day) metatarsals and cultured them in organ culture media containing 200 ng/ml of FGF19 and/or β Klotho (β Klotho (KLB), an essential membrane-bound co-receptor that facilitates FGF19-FGFR binding 25) for 7 days (**Figure 1a**). By using a stereomicroscope, we detected the changes in bone length induced by FGF19 (**Figure 1b**). The results revealed that, after 7 days of organ culture, FGF19 alone did not significantly alter metatarsal growth, whereas co-treatment with KLB led to a marked reduction in bone elongation. These findings were corroborated by measurements of total bone length increase (**Figure 1c**) and mineralized region length (**Figure 1d**). Since bone growth is closely associated with the width of the proliferative zone and the volume of hypertrophic chondrocytes within the growth plate 44, 45, we subsequently investigated the parameters of these critical subregions. By using HE and safranin O staining, we found that the widths of the proliferative zone (PZ) and hypertrophic zones (HZ) in the metatarsal growth plate induced by FGF19 in the presence of KLB were significantly shorter than those in the KLB-only group, whereas there were no significant differences in the resting zones (RZ) (**Figure 1e**), and quantitative analysis of the zonal lengths confirmed these changes (**Figure 1f**). Moreover, the accumulation of bulk proteoglycans in the whole growth plate was also lower in the FGF19+ KLB group than in the KLB group (Safranin O staining in **Figure 1e** and quantification in **Figure S1**). To better characterize the endogenous expression patterns of FGF19, KLB, and FGFR4 in the metatarsal growth plate, we performed immunofluorescence staining on metatarsals from embryonic day 16 (E16), postnatal day 0 (P0), and postnatal day 7 (P7) mice. Endogenous FGF19 was expressed throughout the growth plate, with the highest levels observed in the proliferative zone. KLB was detected at low levels in the resting and proliferative zones but was almost absent in the hypertrophic zone. FGFR4 was expressed across the growth plate, with relatively higher expression in the resting and proliferative zones (**Figure S2**). As the positive rates of Ki67, a cell proliferation antigen 46, and proliferating cell nuclear antigen (PCNA), a central regulator of genome replication 47, represent the active level of cell proliferation, we used immunofluorescence to detect changes in the proliferative capacity of chondrocytes (**Figure 1g**). The results revealed that these two markers were strongly expressed in the PZ and lowly expressed in the RZ, which was consistent with the distribution in cartilage layer 11, 48, 49. Moreover, the expression of Ki67 and PCNA was significantly lower, especially in the PZ (white box), in the FGF19+KLB groups than in the KLB groups. Quantitative analysis of Ki67-positive and PCNA-positive cells confirmed these results (**Figure 1h**). To further identify the direct targeted cells expressing FGF19 in the growth plate, we performed immunofluorescence staining. The results revealed that FGF19 was enriched in both the proliferative and hypertrophic zones after exogenous FGF19 treatment (**Figure S3**), suggesting that exogenous FGF19 potentially acts on chondrocytes in both regions. Together with our previous findings that FGF19 induces cell cycle G2/M arrest in chondrocytes 23, these data collectively support that FGF19 directly inhibits proliferation in the proliferative zone and also affects hypertrophic chondrocyte maturation.

**Figure 2:**
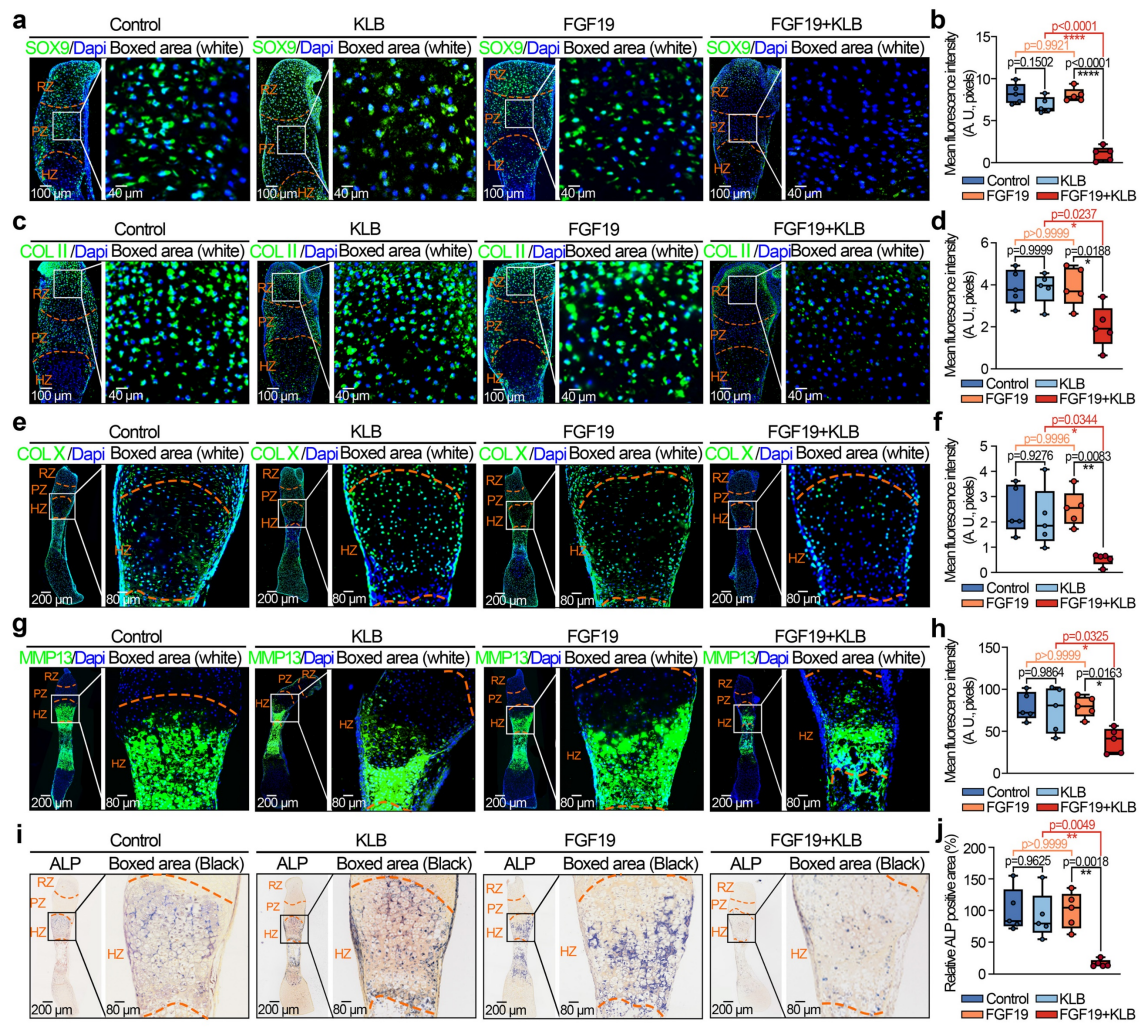
FGF19 reduces chondrocyte maturation and differentiation during the early development of the metatarsal growth plate *ex vivo*.** a.** Immunofluorescence staining showing the changes in SOX9 in the cartilage of the metatarsal bone head induced by FGF19 and KLB for 7 days.** b.** Quantitative analysis of changes in SOX9 expression in metatarsal growth plates.** c.** Immunofluorescence staining showing the changes in COL II in the growth plate of metatarsal bones induced by FGF19 and KLB for 7 days.** d.** Quantitative analysis of changes in COL II expression in metatarsal growth plates.** e.** Immunofluorescence staining showing the changes in COL X in the growth plate of metatarsal bones induced by FGF19 and KLB for 7 days. **f.** Quantitative analysis of changes in COL X expression in metatarsal growth plates. **g.** Immunofluorescence staining showing the changes in MMP13 in the growth plate of metatarsal bones induced by FGF19 and KLB for 7 days. **h.** Quantitative analysis of changes in Mmp13 expression in metatarsal growth plates. **i.** ALP staining showing the changes in ALP activity in the growth plate of metatarsal bones induced by FGF19 and KLB for 7 days. **j.** Quantitative analysis of changes in ALP expression in metatarsal growth plates. The data in **b, d, f, h** and **j** are presented as box (from 25, 50 to 75%) and whisker (standard deviation, SD). The significance analysis in **b, d, f, h** and **j** was based on two-tailed Student's t tests. All the results in **i, c, e, g** and **i** were obtained from at least five independent experiments (n ≥ 5).

To further investigate the influence of FGF19 on chondrocyte maturation and differentiation, we analysed the changes in chondrogenic markers in the metatarsal growth plate. As SOX9 is a critical transcription factor that regulates chondrocyte differentiation and cartilage matrix synthesis (e.g., collagen type II (COL II) and proteoglycans) 50, 51, we detected changes in SOX9 and COL II by immunofluorescence (**Figure 2a-2d**). We found that the levels of SOX9 and COL II in the growth plate induced by FGF19 with KLB were much lower than those in the KLB-only groups (especially the changes in SOX9 in the PZ of the growth plate, boxed areas in **Figure 2a**, and the changes in COL II in the RZ of the growth plate, boxed areas in **Figure 2c**). Quantitative analyses further confirmed the changes in SOX9 (**Figure 2b**) and COL II (**Figure 2d**) induced by FGF19. Because of the crucial role of endochondral osteogenesis in the development of long bones 52, we then focused on cartilage hypertrophy and new bone formation (**Figure 2e-2j**). Immunofluorescent staining revealed that collagen type X (COL X), a well-established marker of hypertrophic chondrocytes 53, was notably reduced in the FGF19+KLB group versus controls (**Figure 2e & 2f**). Similarly, matrix metalloproteinase 13 (MMP13), which is associated with cartilage matrix remodeling during chondrocyte hypertrophy 54, was also downregulated in response to FGF19 treatment (**Figure 2g & 2h**). We next detected new bone formation via alkaline phosphatase (ALP) staining (**Figure 2i**), and the results revealed that FGF19 with KLB significantly reduced ALP-positive areas at the bony end of the metatarsal growth plate. Quantitative analyses further confirmed the changes in ALP-positive areas (**Figure 2j**). Taken together, these results indicate that FGF19, in a KLB-dependent manner, not only reduces early chondrocyte maturation of the metatarsal growth plate but also hinders the maturation of hypertrophic chondrocytes, resulting in blockage of the overall development of the metatarsal bone.

### FGF19 regulates growth plate growth and adolescent bone development

**Figure 3:**
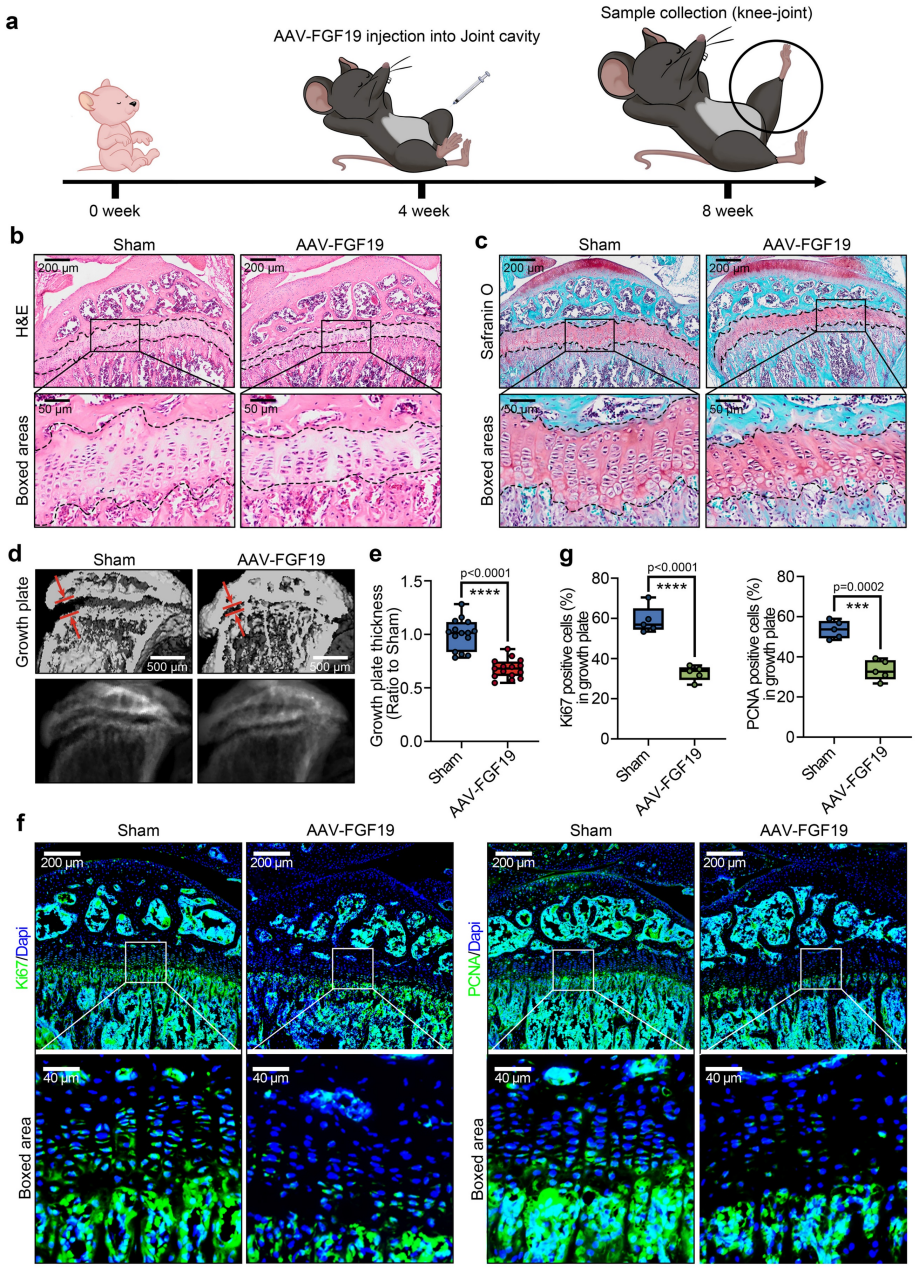
FGF19 overexpression reduces growth plate thickness and reduces chondrocyte proliferation *in vivo*.** a.** Schematic diagram showing the model of FGF19 overexpression via adeno-associated virus (AAV) and sample collection.** b.** H&E staining showing changes in the thickness of the growth plate in AAV-FGF19-overexpressing articular cartilage.** c.** Safranin O staining showing changes in the thickness of the growth plate in AAV-FGF19-overexpressing articular cartilage.** d.** μ-CT results showing changes in the width of the growth plate in AAV-FGF19-overexpressing articular cartilage at 8 weeks.** e.** Quantitative analysis of the width of growth plate cartilage in **d** in AAV-FGF19-overexpressing mice.** f.** Immunofluorescence showing the expression of Ki67 and PCNA in the growth plate cartilage of AAV-FGF19-overexpressing mice.** g.** Quantitative analysis of the percentage of Ki67- and PCNA-positive cells in the growth plate cartilage of AAV-FGF19-overexpressing mice. The data in **e** and **g** are presented as box (from 25, 50 to 75%) and whisker (standard deviation, SD). The significance analysis in **e** and **g** was based on two-tailed Student's t tests. All the results in **b, c, d** and **f** were obtained from at least five independent experiments (n ≥ 5).

To further verify the effect of FGF19 on long bone development *in vivo*, we established an FGF19 overexpression model by using an adeno-associated virus (AAV) carrying the FGF19 gene with the same method described 55. We injected AAV-FGF19 into the knee articular cavity of 4-week-old mice, and the samples were collected at 4 weeks postinjection (**Figure 3a**, 8-week-old samples). As the growth plate of adolescents continues to provide a precise spatiotemporal necessity for long bone growth, the characteristics, including zonal cell integrity, morphology and activity in the growth plate, are crucial for osteogenesis and mineralization 44. By HE and safranin O staining (**Figure 3b & 3c**), we firstly found that the thickness of the tibial growth plate was significantly thinner in the AAV-FGF19 groups than in the sham groups. Importantly, the number and length of cluster-like mature chondrocytes in the growth plate region were significantly shorter in the AAV-FGF19 groups than in the sham groups (indicated by the boxed areas). Quantification about the length of the cluster-like mature chondrocytes in the growth plate region further confirmed these results (**Figure S4**). By μ-CT analysis, we further observed that the thickness of the tibial growth plate was substantially thinner in the AAV-FGF19 group than in the sham group (red arrows, **Figure 3d**). Quantitative analysis further confirmed the thickness change in the growth plate induced by FGF19 (**Figure 3e**). To better characterize the expression dynamics of FGF19 and its receptors at different postnatal developmental stages, we performed immunofluorescence staining on the knee joints of 4- and 8-week-old mice, and found that endogenous FGF19 was broadly expressed across the entire growth plate at both 4- and 8-week-old mice, while KLB and FGFR4 were weakly expressed but still detectable (**Figure S5** and **Figure S6-**sham group). These results suggest that the FGF19 signaling remains active from early postnatal development to adolescence. Furthermore, immunofluorescence analysis at 8 weeks revealed the increased expression levels of FGF19, KLB, and FGFR4 in the growth plates of the AAV-FGF19 overexpression group compared with the sham group (**Figure S6**), indicating successful activation of the FGF19 signaling pathway following AAV-mediated gene delivery. Given that FGF19 impacts the longitudinal growth of the nascent growth plate, as shown in **Figure 1g & 1h**, we next detected the proliferative capacity of adolescent growth plate chondrocytes via immunofluorescence (**Figure 3f**). The results revealed that the expression of Ki67 (left) and PCNA (right) was significantly lower in the AAV-FGF19 groups than in the sham groups. Quantitative analysis of Ki67-positive and PCNA-positive cells further confirmed these results (**Figure 3g**). Collectively, these results suggest that FGF19 overexpression reduces growth plate thickness and decreases chondrocyte proliferation.

**Figure 4:**
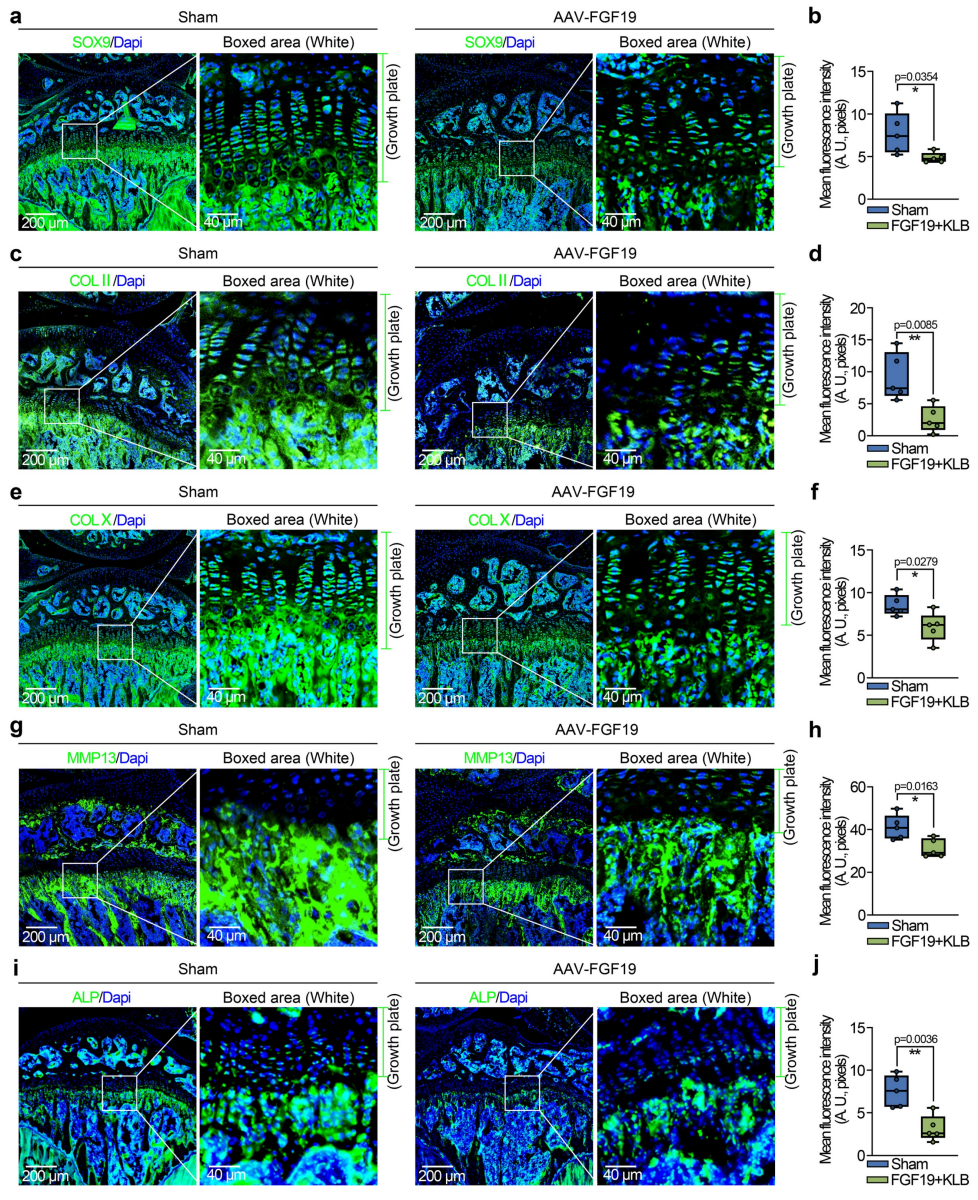
FGF19 overexpression reduces chondrocyte maturation and differentiation *in vivo*. **a.** Immunofluorescence staining showing the changes in SOX9 in the growth plate of AAV-FGF19-overexpressing articular cartilage at 8 weeks. **b.** Quantitative analysis of changes in SOX9 expression in the growth plate of AAV-FGF19-overexpressing articular cartilage. **c.** Immunofluorescence staining showing changes in COL II in the growth plate of AAV-FGF19-overexpressing articular cartilage at 8 weeks. **d.** Quantitative analysis of changes in COL II expression in the growth plate of AAV-FGF19-overexpressing articular cartilage. **e.** Immunofluorescence staining showing the changes in COL X in the growth plate of AAV-FGF19-overexpressing articular cartilage at 8 weeks. **f.** Quantitative analysis of changes in COL X expression in the growth plate of AAV-FGF19-overexpressing articular cartilage. **g.** Immunofluorescence staining showing changes in MMP13 in the growth plate of AAV-FGF19-overexpressing articular cartilage at 8 weeks. **h.** Quantitative analysis of changes in Mmp13 expression in the growth plate of AAV-FGF19-overexpressing articular cartilage. **i.** Immunofluorescence staining showing changes in ALP in the growth plate of AAV-FGF19-overexpressing articular cartilage at 8 weeks. **j.** Quantitative analysis of changes in ALP expression in the growth plate of AAV-FGF19-overexpressing articular cartilage. The data in **b, d, f, h** and **j** are presented as box (from 25, 50 to 75%) and whisker (standard deviation, SD). The significance analysis in **b, d, f, h** and **j** was based on two-tailed Student's t tests. All the results in **a, c, e, g** and **i** were obtained from at least five independent experiments (n ≥ 5).

To further investigate the influence of FGF19 overexpression on chondrocyte maturation and differentiation, we detected changes in the chondrocyte phenotype. In mature chondrocytes, we found that the expression of SOX9 and COL II was significantly lower in the AAV-FGF19 groups than in the sham groups, as shown by immunofluorescence (**Figure 4a & 4c**). Quantitative analysis of the mean fluorescence intensity confirmed the changes in the expression of SOX9 (**Figure 4b**) and COL II (**Figure 4d**). Additionally, immunofluorescence co-staining of FGF19 and SOX9 in the growth plate of AAV-FGF19-treated mice further revealed that overexpressed FGF19 was predominantly distributed in the proliferative zone chondrocytes, where it coincided with a marked reduction in SOX9 expression (**Figure S7a**). Although the spatial localization of FGF19 and the reduction in SOX9 expression suggest that FGF19 may impact the proliferation of chondrocytes by suppressing SOX9, further studies using lineage tracing or conditional knockout models are needed to elucidate the precise mechanism. Reports have shown that chondrocytes can increase their cell volume more than 10-fold during hypertrophy, and this cellular enlargement increases the space for longitudinal bone growth in adolescents 44. To assess the influence of FGF19 on this maturation process in adolescent growth plate chondrocytes, we examined the expression of hypertrophic markers including COL X, MMP13, and ALP. Our findings indicated that FGF19 overexpression notably suppressed the expression of COL X (**Figure 4e**), MMP13 (**Figure 4g**) and ALP (**Figure 4i**) in the hypertrophic cartilage region. These observations were further substantiated through quantitative analysis (**Figure 4f, 4h, 4j**). Consistently, co-staining of FGF19 and MMP13 in the AAV-FGF19 group showed increased FGF19 signal in the hypertrophic zone and adjacent subchondral bone, accompanied by reduced MMP13 expression (**Figure S7b**), suggesting that FGF19 may also impair hypertrophic chondrocyte maturation and endochondral bone formation. Taken together, these results suggest that FGF19 overexpression *in vivo* inhibits the maturation and hypertrophic differentiation of growth plate chondrocytes.

### FGF19 mediates chondrogenesis and the chondrocyte phenotype

**Figure 5:**
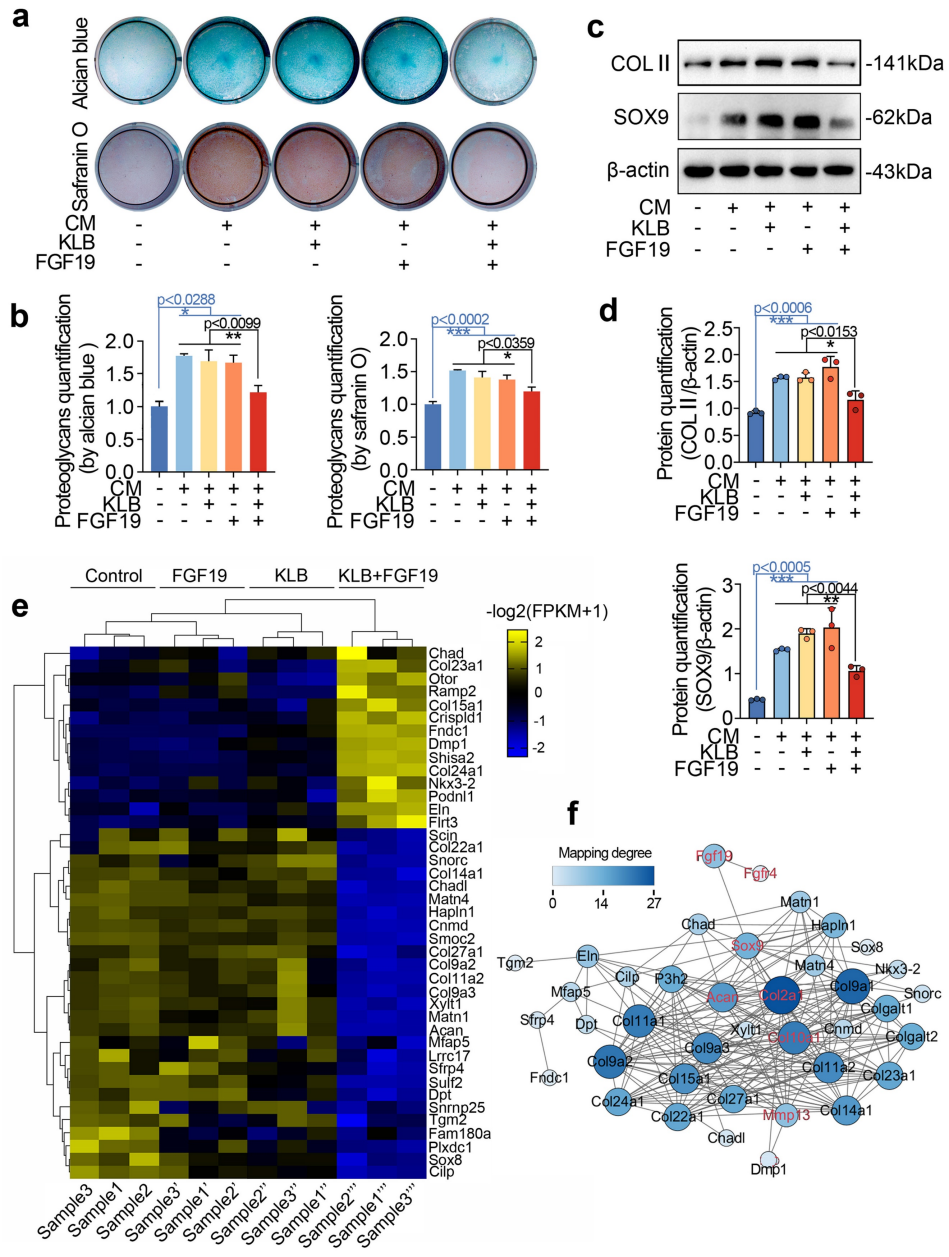
FGF19 reduces chondrogenic differentiation *in vitro*. **a.** Alcian blue and Safranin O staining showing the accumulation of proteoglycan production in BMSCs induced by FGF19 in the presence of KLB. Confluent BMSCs seeded in 24-well plates were cultured in 2% FBS αMEM and CM for 7 days before staining. CM, chondrogenic medium. **b.** Quantitative analysis of the accumulation of proteoglycans in (a). **c.** Western blot showing the COL II and SOX9 levels in BMSCs stimulated with FGF19 (200 ng/ml) in the presence of KLB (200 ng/ml). **d.** Quantitative analysis of the COL II and SOX9 levels in (c). **e.** RNA sequencing showing the changes in the cartilage phenotype and collagen extracellular matrix in chondrocytes induced by FGF19 (200 ng/ml) in the presence of KLB (200 ng/ml). **f.** Protein‒protein interaction network based on clustered genes in (e) showing the impact of FGF19 on chondrogenesis and the chondrocyte phenotype. The data in **b** and** d** are presented as the means ± SD. The significance analysis in **b** and** d** was based on two-tailed Student's t tests. All the results in **a** and** c** were obtained from three independent experiments (n = 3).

To further reveal the role of FGF19 in chondrogenesis, we conducted *in vitro* differentiation assays using bone marrow mesenchymal stem cells (BMSCs) under chondrogenic induction. With the induction of chondrogenic culture media for 7 days, we showed that FGF19 reduced the accumulation of proteoglycans in differentiated BMSCs in the presence of KLB, as shown by alcian blue (upper) and safranin O (lower) staining (**Figure 5a & 5b**). Consistent with this, western blot analysis revealed a significant decline in COL II and SOX9 protein levels in the FGF19+KLB group (**Figure 5c**), which was confirmed by densitometric quantification (Figure 5d). To further validate these changes at the transcriptional level, we performed q-PCR analysis, and found that FGF19 reduced mRNA levels of chondrogenic markers including *Col2a1* and *Sox9* with the help of KLB (**Figure S8**). To further reveal the role of FGF19 in the phenotype and differentiation of chondrocytes, we investigated transcriptome changes in neonatal chondrocytes via RNA sequencing. By a pheatmap, we clustered the gene profiles associated with the chondrocyte phenotype and extracellular matrix components of chondrocytes induced by FGF19 at 200 ng/ml in the presence of KLB **(Figure 5e)**. The data revealed that many genes that mediate extracellular matrix homeostasis and chondrocyte differentiation, including *Acan*, *Matns*, *Cnmd*, *Col27a1*, *Col9*, *Sox8* and *Cilp*, were significantly downregulated, indicating that FGF19 impaired the chondrocyte phenotype and matrix integrity in the presence of KLB. We next established a protein‒protein interaction network to indicate the impact of FGF19 on chondrogenesis and chondrocyte phenotype based on these changed gene candidates (**Figure 5f**), and the results revealed that FGF19 negatively regulated the chondrogenesis and chondrocyte phenotype, especially with respect to the changes in SOX9, ACAN, COL2Α1, COL10Α1, and MMP13.

### FGF19 regulates chondrogenic differentiation via β-catenin signalling

**Figure 6:**
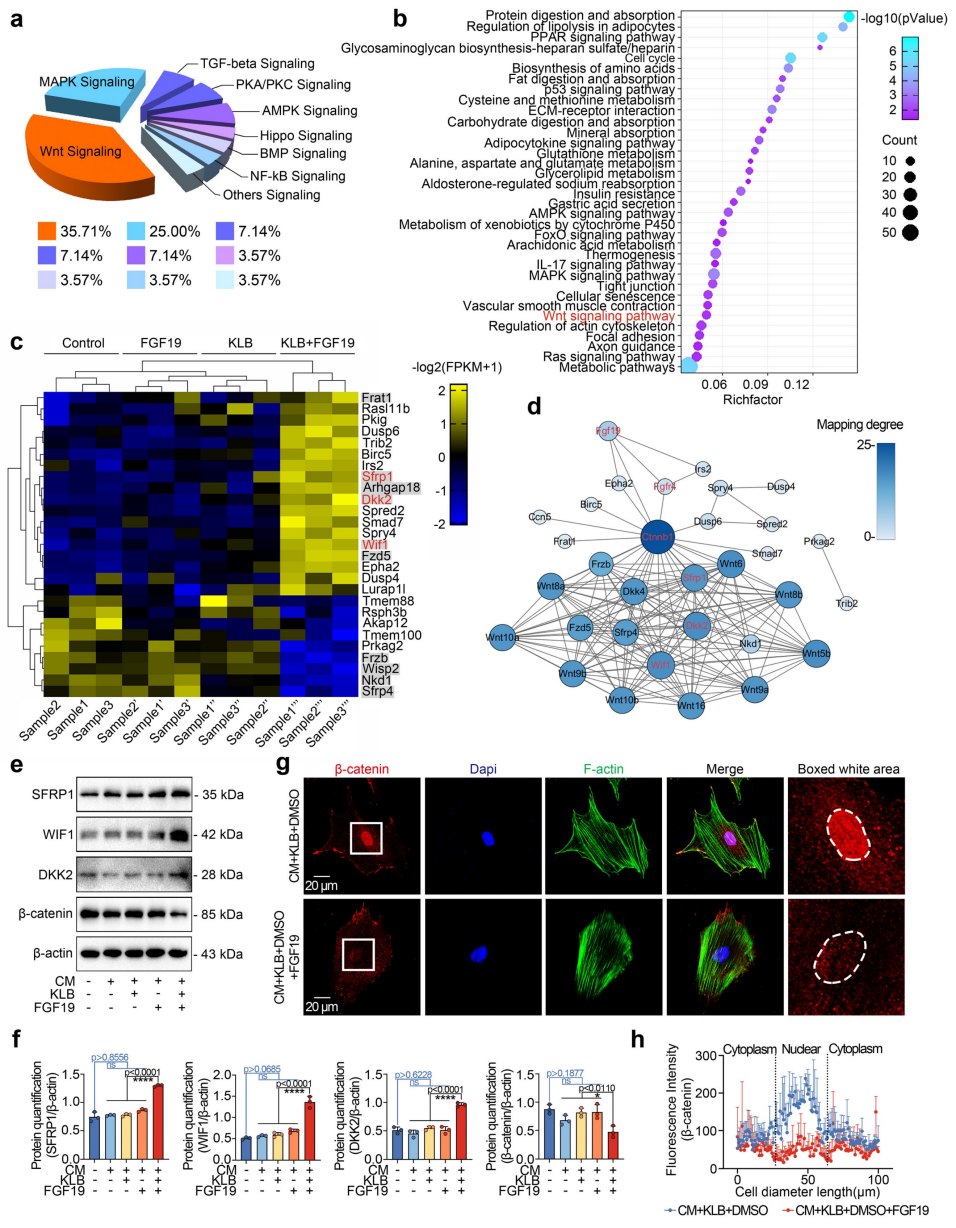
FGF19 induces changes in the Wnt/β-catenin signalling pathway. **a.** Pie chart showing the specific proportions of enriched signalling pathways. **b.** KEGG analysis based on RNA sequencing data showing that high proportions of the targets that interact with FGF19. **c.** Heatmap showing the relative gene changes in the Wnt signalling pathway based on RNA sequencing. **d.** Protein‒protein interactions showing the interaction network of Wnt signalling pathways surrounding these candidates, including SFRP1, WIF1 and DKK2, induced by FGF19. **e.** Representative western blots showing that FGF19 (200 ng/ml) increased the levels of SFRP1, WIF1, and DKK2 and decreased the level of β-catenin in chondrocytes in the presence of KLB (200 ng/ml). **f.** Quantitative analysis of the changes in the levels of SFRP1, WIF1, DKK2 and β-catenin shown in (e). **g.** Representative immunofluorescence images showing the reduced nuclear localisation of β-catenin in BMSCs induced by FGF19 with KLB. Cytoskeleton (F-actin), green; β-catenin, red; Nucleus (DAPI), blue. **h.** Quantification of the linear fluorescent protein β-catenin showing the intracellular distribution of β-catenin in the BMSCs in (g). The data in **f** and **h** are presented as the means ± SD. The significance analysis in **f** was based on two-tailed Student's t tests. All the results in **e** and **g** were obtained from three independent experiments (n = 3).

We next analysed the signalling pathways in chondrocytes induced by FGF19 at the transcriptional level. RNA sequencing revealed that a series of signalling pathways were activated, and most gene clusters were involved in the Wnt signalling (**Figure 6a**). A bubble chart generated via KEGG analysis also revealed the direct participation of Wnt signalling (**Figure 6b**). We clustered the top 27 candidate genes related to cytoplasmic signalling and found that 10 of them belong to the Wnt signalling pathway, i.e., *Dkk2*, *Wif1*, *Frat1*, *Arhgap18*, *Fzd5*, *Sfrp1*, *Frzb*, *Nkd1*, *Wisp2* and *Sfrp4* (**Figures 6c & S9**). Furthermore, we observed that the upregulated genes are Wnt/β-catenin antagonists, including *Sfrp1*, *Wif1*, and *Dkk2* (red), indicating the inactivation of canonical β-catenin signalling. We then established protein‒protein interactions (**Figure 6d**) and constructed an interaction network of Wnt signalling pathways surrounding these changed candidates induced by FGF19. We subsequently used western blotting to verify the protein expression changes of SFRP1, DKK2, WIF1, and β-catenin in chondrocytes treated with FGF19 in the presence of KLB. The data demonstrated that FGF19 upregulated the expression of SFRP1, WIF1, and DKK2, while downregulating β-catenin levels (**Figure 6e**). These alterations were further confirmed by quantitative analysis (**Figure 6f**). To further validate these changes at the transcriptional level, we performed q-PCR analysis. The results showed that the mRNA levels of *Sfrp1*, *Wif1*, and *Dkk2* were upregulated, while *Ctnnb1* was downregulated upon FGF19 and KLB treatment, which was consistent with the protein expression trends observed above (**Figure S10**). Canonical Wnt/β-catenin signalling is activated when β-catenin dissociates from the destruction complex composed of glycogen synthase kinase 3 (GSK-3) and casein kinase 1 (CK1), allowing it to translocate into the nucleus and initiate transcription of downstream target genes 56. To investigate how FGF19 affects the subcellular distribution of β-catenin, we conducted immunofluorescence assays (**Figure 6g**). The results showed that FGF19, in the presence of KLB, markedly reduced β-catenin levels, particularly in the nucleus (highlighted by white circles). Linear fluorescence quantification confirmed the changes in the nuclear accumulation of β-catenin (**Figure 6h**), and fluorescence quantification confirmed the level of intracellular β-catenin in individual cells (**Figure S11**). Collectively, these results indicate that FGF19 negatively regulates the activation of β-catenin signalling in chondrocytes.

**Figure 7:**
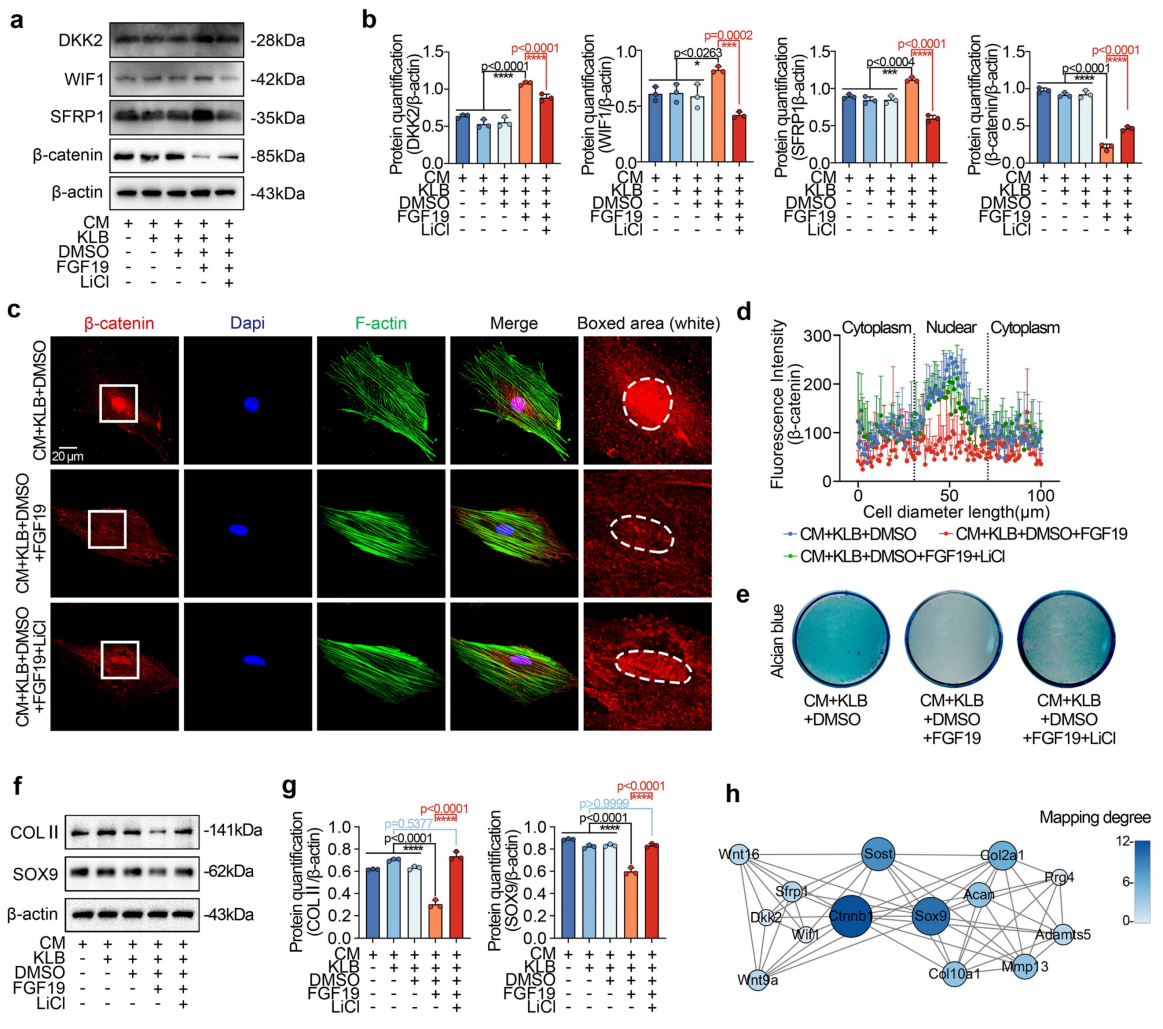
FGF19 impairs chondrogenic differentiation through the Wnt/β-catenin signalling pathway. **a.** Representative western blots showing that LiCl inhibited the changes in the levels of Wnt antagonists (SFRP1, DKK2 and WIF1) and reversed the decrease in the level of β-catenin. **b.** Quantitative analysis of the changes in the levels of SFRP1, WIF1, DKK2 and β-catenin shown in (a). **c.** Representative immunofluorescence images showing the increase in β-catenin in BMSCs after pretreatment with 20 mM LiCl in the presence of FGF19 and KLB. Cytoskeleton (F-actin), green; β-catenin, red; Nucleus (DAPI), blue. **d.** Quantification of the linear fluorescence intensity of β-catenin in the BMSCs shown in (c). **e.** Alcian blue staining revealed that LiCl restored the loss of proteoglycan production in BMSCs induced by FGF19 in the presence of KLB. **f.** Representative western blots showing that FGF19 induced changes in the protein levels of COL II and SOX9 in chondrogenic BMSCs in the presence or absence of LiCl. **g.** Quantification analysis to confirm the protein changes in (f). **h.** Protein‒protein interaction network showing that FGF19 mediate chondrogenic differentiation by regulating β-catenin signalling. The data in **b, d** and **g** are presented as the means ± SD. The significance analysis in **b** and** g** was based on two-tailed Student's t tests. All the results in **a, c, e** and **f** were obtained from three independent experiments (n = 3).

To further confirm the negative regulation of β-catenin signalling in chondrogenic differentiation induced by FGF19, we applied the chemical reagent LiCl, a β-catenin-specific activator 57. By western blotting, we found that LiCl could partially restore the FGF19-induced decrease in β-catenin and reduce the levels of SFRP1, DKK2, and WIF1 in chondrogenic BMSCs induced by FCF19 (**Figures 7a & 7b**). We then performed immunofluorescence to detect the changes in β-catenin in chondrogenic BMSCs induced by LiCl in the presence of FGF19 and KLB (**Figure 7c**). The immunofluorescence results indicated that LiCl partially rescued β-catenin nuclear accumulation under these conditions. Quantitative analyses of fluorescence intensity supported this observation (**Figures 7d & S12**). After 7 days of chondrogenic induction, Alcian blue staining (**Figure 7e**) showed that LiCl also reversed the reduction in proteoglycan accumulation caused by FGF19+KLB, with quantitative confirmation provided in **Figure S13**. Additionally, LiCl treatment increased the protein levels of COL II and SOX9 in the FGF19+KLB context, as confirmed by western blotting (**Figures 7f & 7g**). Taken together, these results indicate that FGF19 mediates chondrogenic differentiation by negatively regulating β-catenin-SOX9 axis (**Figure 7h**).

### FGF19-regulated chondrocyte maturation requires the participation of FGFR4

**Figure 8:**
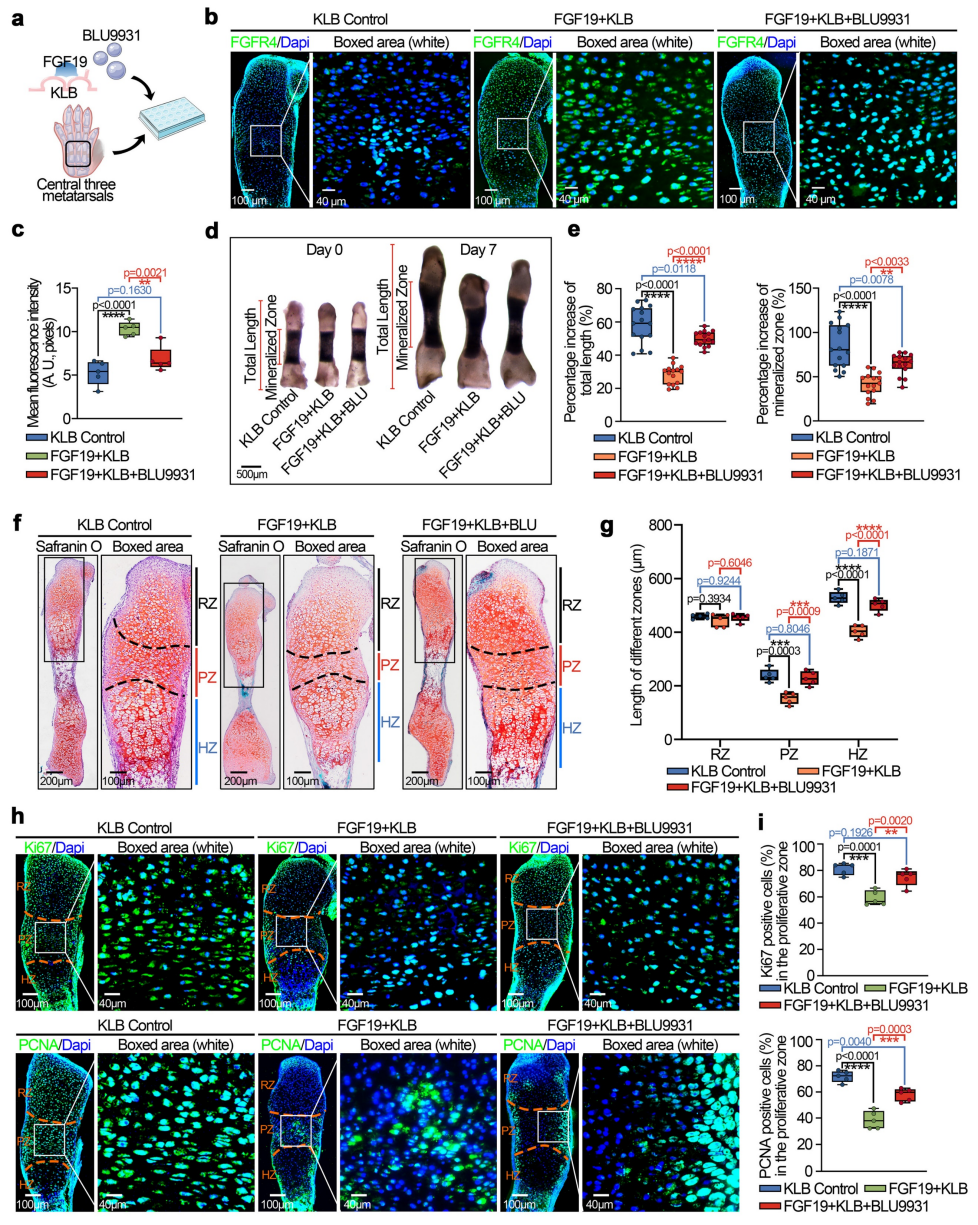
FGF19 reduces metatarsal longitudinal growth and growth plate chondrocyte proliferation via FGFR4 *ex vivo*. **a.** Schematic diagram showing the model of *ex vivo* metatarsal organ culture induced by FGF19 (200 ng/ml), KLB (200 ng/ml) and BLU9931 (5 μM). **b.** Immunofluorescence showing the changes in the abundance of FGFR4 in the metatarsal growth plate induced by FGF19 in the presence of BLU9931. **c.** Quantitative analysis of changes in FGFR4 expression in metatarsal growth plates. **d.** Representative stereomicroscope images showing that BLU9931 restored the length of the metatarsal bone induced by FGF19 and KLB. **e.** Quantitative analysis of the percentage growth of the total length and mineralized region of the metatarsal bones in (d). **f.** Safranin O staining showing the length changes in the RZ, PZ, and HZ of the growth plate in the metatarsal bone head induced by BLU9931 in the presence of FGF19 and KLB. **g.** Quantitative analysis of the lengths of the RZ, PZ, and HZ of the growth plate in the metatarsal bone head. **h.** Immunofluorescence staining showing the level and distribution of Ki67 and PCNA in the metatarsal growth plate induced by BLU9931 in the presence of FGF19 and KLB. **i.** Quantitative analysis of the percentage of Ki67- and PCNA-positive cells in metatarsal growth plates. The data in **c, e, g** and **i** are presented as box (from 25, 50 to 75%) and whisker (standard deviation, SD). The significance analysis in **c, e, g** and **i** was based on two-tailed Student's t tests. All the results in **b, d, f** and **h** were obtained from at least five independent experiments (n ≥ 5).

FGF19 is known to bind preferentially to FGFR4 in the presence of KLB 27. Our previous studies also demonstrated the importance of FGFR4 in cell cycle regulation and mitochondrial metabolism in chondrocytes 23, 33. In this study, we observed an upregulation of FGFR4 expression in the FGF19-treated group compared to the KLB-only group (**Figure S14**, two & three lanes) and also in the AAV-FGF19-injected group relative to the sham control (**Figure S6**). To further validate the role of FGFR4 in FGF19-mediated chondrocyte maturation and bone development, we applied the chemical regent BLU9931 (**Figure 8a**), a specific inhibitor of FGFR4^58^.

We first investigated the expression of FGFR4 in chondrocytes pretreated with BLU9931 in the presence of FGF19 and KLB *in vitro* (**Figure S14,** lower two lanes) and in cartilage *ex vivo* by immunofluorescence (**Figure 8b & 8c**). The results indicated that BLU9931 could reduce the expression of FGFR4 enhanced by FGF19. Morphological analysis by stereomicroscopeoly (**Figure 8d**) revealed that the bone length in the BLU9931 group was close to that in the KLB control group, which indicates the blockage of FGFR4 reduced the influence of FGF19 in the nascent bone development. Quantitative analysis of the lengths of the total bone and mineralized region confirmed these results (**Figure 8e**). Safranin O staining revealed that the lengths of the proliferative and hypertrophic zones in the BLU9931 group were close to those in the KLB control group (**Figures 8f & 8g**). Additionally, the accumulation of bulk proteoglycans was higher in the BLU9931 group than in the FGF19 control group (**Figure 8f & S15**). Finally, we explored the proliferative capacity via immunofluorescence (**Figure 8h**). We found that the expression of Ki67 (upper) and PCNA (lower) was partially restored in the BLU9931 group compared with the FGF19 control groups. Quantitative analysis of Ki67-positive (upper) and PCNA-positive (lower) cells confirmed these results (**Figure 8i**). Overall, these results indicate that FGF19-mediated chondrocyte maturation and nascent bone development require the participation of FGFR4.

### FGF19 relies on FGFR4 to negatively regulate β-catenin signalling

**Figure 9:**
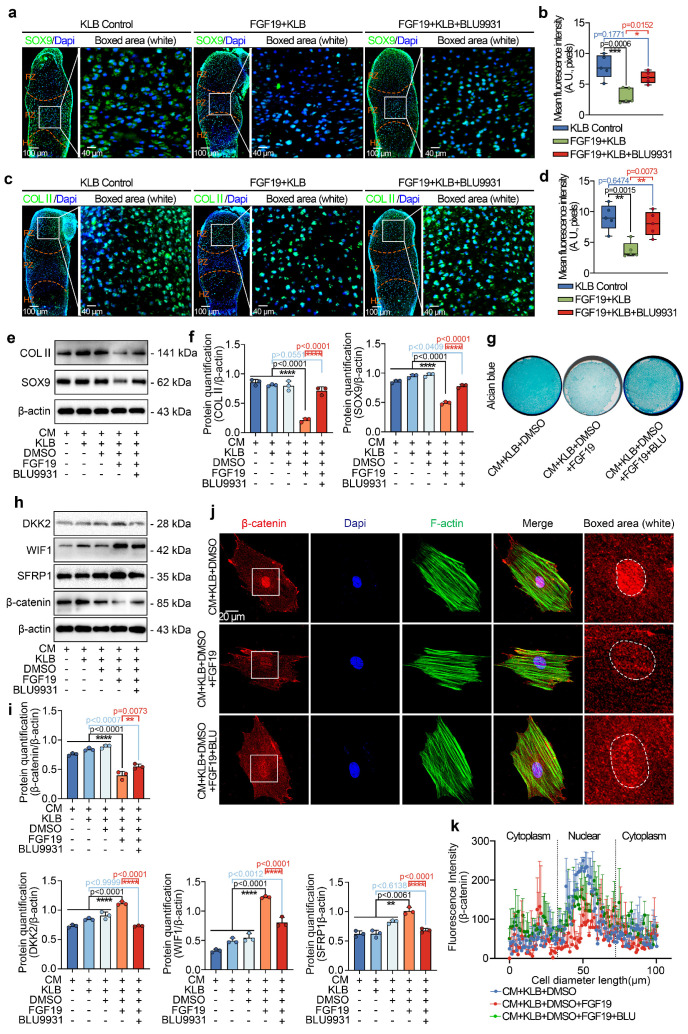
FGF19 impairs chondrocyte maturation and differentiation through the FGFR4/Wnt/β-catenin axis. **a.** Immunofluorescence staining showing the changes in SOX9 in the growth plate of the metatarsal bone head induced by BLU9931 in the presence of FGF19 and KLB. **b.** Quantitative analysis of changes in SOX9 expression in metatarsal growth plates. **c.** Immunofluorescence staining showing the changes in COL II in the growth plate of the metatarsal bone head induced by BLU9931 in the presence of FGF19 and KLB. **d.** Quantitative analysis of changes in COL II expression in metatarsal growth plates. **e.** Representative western blots showing the levels of COL II and SOX9 in chondrogenic BMSCs treated with FGF19 and KLB in the presence or absence of BLU9931. **f.** Quantification of COL II and SOX9 protein levels in (e). **g.** Alcian blue staining revealed that BLU9931 reversed the reduction in proteoglycan production in chondrogenic BMSCs induced by FGF19 and KLB. **h.** Representative western blots showing that FGF19 combined with KLB induced changes in the levels of SFRP1, WIF1, DKK2 and β-catenin in chondrogenic BMSCs in the presence or absence of BLU9931. **i.** Quantification of SFRP1, WIF1, DKK2 and β-catenin protein expression in** (**h). **j.** Representative immunofluorescence images showing the nuclear accumulation of β-catenin in chondrogenic BMSCs induced by BLU9931 in the presence of FGF19 and KLB. Cytoskeleton (F-actin), green; β-catenin, red; Nucleus (DAPI), blue. **k.** Quantification of the linear fluorescence intensity of β-catenin in the BMSCs shown in (j). The data in **b, d, f, i** and **k** are presented as box (from 25, 50 to 75%) and whisker (standard deviation, SD). The significance analysis in **b, d, f** and **i** was based on two-tailed Student's t tests. The results in **a** and **c** were obtained from at least five independent experiments (n ≥ 5), and the results in **e, g, h** and** j** were obtained from three independent experiments (n = 3).

To further confirm the importance of FGFR4 in cartilage development induced by FGF19, we investigated the protein levels of SOX9 and COL II via immunofluorescence *ex vivo* (**Figure 9a-9d**). From SOX9 staining, we found that BLU9931 could partially restore the expression of SOX9 in the metatarsal growth plate in the presence of FGF19 and KLB (**Figure 9a & 9b**), meanwhile BLU9931 could also restore the expression of COL II in the metatarsal growth plate in the presence of FGF19 and KLB (**Figure 9c & 9d**). By western blotting, we found that the abundance of SOX9 and COL II in chondrogenic BMSCs treated with BLU9931 in the presence of FGF19 was largely restored relative to that in the FGF19 group (**Figure 9e & 9f**). After chondrogenic induction for 7 days, alcian blue staining revealed that BLU9931 increased the accumulation of proteoglycans in BMSCs in the presence of FGF19 and KLB (**Figure 9g & S16**). These results revealed the importance of FGFR4 in cartilage maturation induced by FGF19.

To further investigate the role of FGFR4 in Wnt/β-catenin signalling in chondrogenic BMSCs induced by FGF19, we next performed western blotting and found that treatment with BLU9931 impaired the increase in the levels of the Wnt antagonists, SFRP1, DKK2, and WIF1 and partially restored the β-catenin level (**Figure 9h**). Quantitative analysis further confirmed the changes in the SFRP1, DKK2, WIF1 and β-catenin levels (**Figure 9i**). Finally, we used immunofluorescence to determine the effect of FGFR4 on the cytoplasmic distribution of β-catenin (**Figure 9j**). BLU9931 treatment restored both total and nuclear β-catenin levels in individual cells. These changes were validated by linear fluorescence measurements (**Figure 9k**) and mean fluorescence intensity quantification at the single-cell level (**Figure S17**). Collectively, these findings confirm the importance of FGFR4 in the canonical β-catenin signalling negatively regulated by FGF19.

## Discussion

As a member of the FGF family, FGF19 is involved in mediating the development of the eyes, ears, brain, heart, and liver 24,59,60. In recent years, increasing attention has been given to its potential functions in skeletal development. The presence of FGF19 in fetal cartilage of the lower limbs and in the femoral growth plate suggests it may play a physiological role in endochondral ossification 28. Research using zebrafish models has shown that FGF19 contributes to craniofacial cartilage formation via the MAPK signalling cascade 61. Our recent research revealed that FGF19 inhibits cell proliferation and mediates G2 phase arrest of the chondrocyte cell cycle 23. Additionally, work by Erika Yeh et al. indicated that the ectopic interaction between FGF19 and FGFR2 can either promote or suppress osteogenic differentiation by modulating the expression of RUNX2 and BMP antagonists 31. Other studies have revealed that FGF19 can enhance skeletal muscle mass and strength in mice and can increase the size of cultured human myotubes by promoting phosphorylation of ERK1/2 and S6K1^62^. In the current study, we elucidate that high levels of FGF19 disrupt growth plate development by inhibiting chondrocyte proliferation, early chondrogenic differentiation, and hypertrophic maturation, thus impairing endochondral osteogenesis.

**Figure 10:**
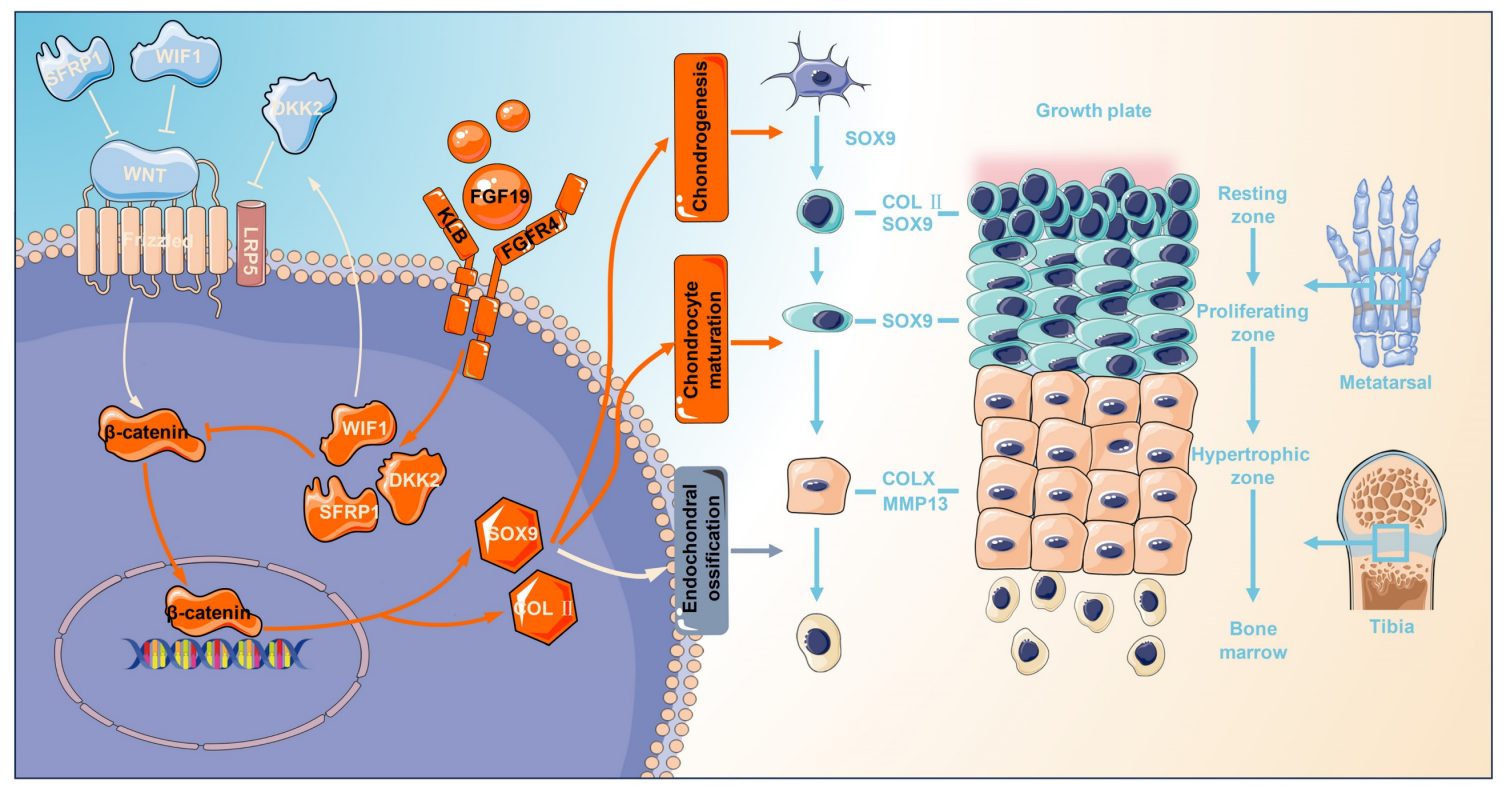
Schematic showing how FGF19 mediates cartilage development. FGF19 impacts the processes from chondrogenesis, chondrocyte maturation, to endochondral ossification. For chondrogenesis, FGF19 inhibited the upregulation of chondrogenic markers such as SOX9 and COL II, thereby suppressing the initiation of chondrogenic differentiation. For chondrocyte maturation, FGF19 predominantly accumulates in the proliferative zone and suppresses SOX9 expression, indicating strong impact on proliferative/matured chondrocytes. For chondrocyte hypertrophy and endochondral ossification, FGF19 decreases the expression of COLX, MMP13, and ALP, inferring the impairment in hypertrophy and endochondral ossification. FGF19 enters chondrocytes through FGFR4 and activates the Wnt/β-catenin antagonists, SFRP1, WIF1, and DKK2, and thus decreases the expression of β-catenin and its nuclear accumulation, resultantly impacting cartilage development.

Endochondral ossification is an essential process in mammalian long-bone growth and is strictly regulated by various transcription factors and growth factors, such as SOX9, RUNX2, TGF-β, BMPs, IGF, and FGFs. TGF-β and BMPs are necessary for SOX9 expression and maintenance and are thus essential for mesenchymal condensation during cartilage formation. TGF-β, BMPs, and their receptors are expressed in proliferating and hypertrophic chondrocytes, inducing chondrocyte proliferation and inhibiting terminal chondrocyte differentiation through common Smad/R-Smad complexes or MAPK cascade transduction 63. TGF-β1 and TGF-β3 are widely used for chondrogenesis and maintenance of chondrogenic phenotypes 6,64. IGF-1 has potent anabolic effects and can promote chondrocyte proliferation, enhance the synthesis of type II collagen and proteoglycans, and promote BMSC chondrogenic differentiation, thus playing a critical role in prenatal cartilage growth 65. IGF-1 is expressed throughout the growth plate, but its expression level is lower than that of IGF-2. IGF-2 is strongly expressed in all regions of the growth plate, particularly in the proliferative zone, which is essential for the longitudinal development of cartilage in the postnatal period 66. FGFs and their corresponding receptors exhibit spatial‒temporal characteristics throughout all phases of cartilage and bone formation. FGFR1 is found in regions such as the condensed mesenchyme, perichondrium, and hypertrophic zones of the growth plate, while FGFR2 is initially detected in the condensed mesenchyme and later becomes expressed in the perichondrium and periosteum 12. FGFR3, on the other hand, is primarily localized in proliferating chondrocytes within the growth plate. This receptor plays a dual role when bound to its ligands, FGF9 and FGF18. In the early stages of development, the binding of FGFR3 to its ligands promotes chondrocyte proliferation and initial chondrocyte hypertrophy; conversely, FGFR3 inhibits chondrocyte proliferation and hypertrophy in the later stages of development 12. FGFR4 is expressed in the resting and proliferative zones, and studies have shown that FGF18 regulates chondrocyte autophagy through FGFR4, thereby modulating the developmental process 22. Our research demonstrated that FGF19 inhibits the proliferation of growth plate chondrocytes and suppresses the expression of chondrocyte differentiation markers (SOX 9 and COL II) and maturation markers (COL X and MMP 13). Furthermore, FGF19 functions by binding to FGFR4 and activating the expression of the Wnt inhibitory factor, thus inhibiting the Wnt/β-catenin pathway. Notably, given the potential crosstalk among endocrine FGFs, it is important to consider their distinct roles in cartilage development. Although high concentrations of FGF21 have been reported to suppress chondrocyte proliferation and differentiation, FGF21 primarily signals through FGFR1-KLB and has low affinity for FGFR4-KLB, making it unlikely to activate FGFR4-KLB under physiological conditions 67,68. Similarly, FGF23 regulates phosphate homeostasis through FGFR3 in the presence of soluble α-Klotho and inhibits linear bone growth by reducing chondrocyte proliferation 69,70. These findings suggest that FGF21 and FGF23 function through distinct receptor complexes from that of FGF19. Therefore, in our model where FGF19 signals through the KLB-FGFR4 axis, the observed effect reflects the role of FGF19 rather than the role of other subfamily members. However, it remains a possible that FGF19 overexpression indirectly impacts the expression or signaling of other endocrine FGFs, thereby influencing chondrogenesis. The role of FGF19 on chondrogenesis and endochondral ossification in the current study enriches the understanding of FGF family on skeletal system development (**Figure 10**), and correlates with the other mutifactors including TGF-β, BMPs, and IGFs, which synergistically regulate the endochondral ossification and bone formation.

Multiple signalling pathways play crucial roles in regulating the progression of endochondral ossification, including IHH, PTHrP, Notch, and Wnt 4,10,71. IHH signalling facilitates mesenchymal stem cell differentiation into chondrocytes and supports their proliferation through a feedback loop with PTHrP, which, in turn, regulates hypertrophic differentiation 4. Notch signalling, expressed in the precartilaginous mesenchyme, inhibits mesenchymal condensation and early chondrocyte differentiation but promotes osteoblast differentiation at later stages 72. Wnt signalling plays a multifaceted role in skeletal development, with the canonical Wnt/β-catenin pathway acting as a key regulator throughout various stages of chondrocyte maturation in the growth plate 73. Suppression of β-catenin activity can lead to aberrantly elevated SOX9 expression, reduced proliferation of chondrocytes, and delayed onset of hypertrophy 71. Conditional inactivation of β-catenin results in a decreased height of the hypertrophic zone in the growth plate, reduced cell proliferation, and delayed cartilage calcification 74. Thus, Wnt/β-catenin signalling is required to regulate growth plate chondrocyte proliferation and differentiation 71. In the present study, RNA sequencing revealed the most substantial alterations in the Wnt/β-catenin signalling pathway after FGF19 stimulation (**Figure 6a**). Further analysis revealed that FGF19 stimulated an increase in the expression of several Wnt inhibitory factors during chondrogenic differentiation (**Figure 6c**), which inhibited Wnt activity by decreasing the β-catenin protein level and nuclear accumulation, resulting in attenuated chondrogenic differentiation of BMSCs (**Figure 7**). SFRP inhibits the Wnt signaling pathway by physically binding to Wnt ligands and their receptor Frizzled 75. WIF1 directly interacts with and inhibits Wnts^76^. DKK1 has been shown to interact with LRP-like Wnt co-receptors, thereby interfering with Wnt signaling 77. Moreover, SFRP1, DKK, and WIF1 have been identified as important regulators of Wnt protein activity in bone development and the maintenance of articular cartilage 78. Numerous *in vitro* studies involving different cell types (including primary chondrocytes, human synovial fluid-derived MSCs, and bone marrow mesenchymal stem cells) have shown that blocking β-catenin significantly hampers chondrogenic differentiation 71, aligning with our observations. Restoration of β-catenin activity through chemical activators was able to reverse the inhibitory effects of FGF19 on chondrocyte differentiation (**Figure 7**). These findings suggest that FGF19 inhibits the Wnt/β-catenin signalling pathway by activating Wnt antagonists to hinder chondrogenic differentiation (**Figure 10**). Our results provide new evidence for the role of Wnt/β-catenin signalling in chondrogenic differentiation *ex vivo*.

Our findings reveal FGF19 exerts its inhibitory effect on cartilage development through activation of FGFR4, which in turn modulates β-catenin signaling. Our data consistently demonstrate that exogenous FGF19, in the presence of its co-receptor KLB, engages FGFR4 in growth plate chondrocytes, as evidenced by increased FGFR4 expression and co-localization in immunofluorescence analysis. This activation leads to the upregulation of Wnt antagonists (SFRP1, WIF1, and DKK2), which suppress nuclear accumulation of β-catenin and reduce the expression of downstream targets critical for chondrogenesis, including SOX9 and COL Ⅱ. These molecular changes correlate with reduced chondrocyte proliferation and impaired longitudinal bone growth in both *ex vivo* and *in vivo* models. To further validate this axis, we confirmed that inhibition of FGFR4 or β-catenin reverses the FGF19-induced suppression of cartilage development. These findings refine our understanding of the FGFR4-specific role among FGFR family members during postnatal cartilage development and may have significant therapeutic implications. Given the inhibitory role of FGF19 in endochondral ossification, targeting the FGF19-FGFR4-β-catenin pathway presents a promising strategy for treating skeletal growth disorders characterized by growth plate dysfunction, such as idiopathic short stature and certain chondrodysplasias. Moreover, since FGF19 signaling may hinder cartilage regeneration, modulating its activity could also benefit the treatment of degenerative joint diseases such as osteoarthritis. Potential interventions may include neutralizing antibodies against FGF19, selective FGFR4 inhibitors, or modulators of downstream Wnt signaling components to enhance chondrogenesis while preserving tissue homeostasis. Collectively, these findings suggest that FGF19 represents a viable target for modulating skeletal growth and cartilage repair.

Our study provides an initial explanation of the role of FGF19 in chondrogenesis, but there are several limitations. Firstly, the *ex vivo* metatarsal culture model has recognised limitations, although chondrogenesis is intact and skeletal growth is impaired. Therefore, metatarsal growth *ex vivo* is approximately 50% slower than that observed *in vivo* 43,79. Although there is a gap from* in vivo* skeletal development, the use of the *ex vivo* metatarsal model is reliable because this study focused only on chondrogenic differentiation. Second, although the *ex vivo* and AAV-FGF19 overexpression models provide valuable insights, they may not fully capture the complexity of *in vivo* conditions. Specifically, genetically modified mice using knockouts of FGF19, KLB, and FGFR4 is essential for confirming the physiological relevance. However, there are technical challenges in generating conventional FGF19-/- and FGFR4-/- mice, as both models exhibit perinatal lethality 80,81, making them unsuitable for studying postnatal skeletal development. Meanwhile, KLB knockout mice show fetal growth restriction prior to placental maturation 82, which further limits their applicability in cartilage-specific investigations and has to consider it as an auxiliary factor of the growth factor FGFs in the most studies. Even so, our results provide an understanding of the importance of FGF19 in the development of cartilage and endochondral ossification in postnatal mice. Thirdly, as we mainly focused on chondrocytes and chondrocyte precursor cells in cartilage, other cell types related to the growth plate such as perichondrocytes, osteoblasts, and endothelial cells can also affect overall bone growth after the induction of FGF19, even if their effects are limited due to their small numbers. This has to be taken into consideration when it comes to cartilage/bone growth. Fourthly, we observed changes in multiple signalling pathways after FGF19 stimulation (**Figure 6a**); however, in the present study, we only demonstrated the importance of Wnt/β-catenin signalling and did not elucidate the roles of other pathways, such as the MAPK signalling pathway. Investigating the roles of other pathways in FGF19-mediated chondrogenesis and endochondral ossification is highly important.

In summary, we found for the first time that FGF19 inhibited chondrocyte proliferation in growth plates, leading to loss of growth plate activity and severe impairment of longitudinal bone growth, and that FGF19 inhibited the expression of early chondrogenic protein markers in the growth plate, as well as inhibited chondrocyte maturation and hypertrophic differentiation. The comprehensive inhibitory/activator effects on receptor binding and signalling activation collectively indicate that FGF19 mediates chondrocyte maturation of the growth plate via the FGFR4/β-catenin signalling axis. This study provides insight into the role of FGF19 in endochondral ossification.

## Supplementary Material

Supplementary figures.
