# Supplementary material for: Fibroblast Growth Factor 19 Disrupts Cartilage Development Via the FGFR4/β-catenin Axis
Source: Int J Biol Sci. 2025 Jul 4;21(10):4428–49. doi: 10.7150/ijbs.110133 (PMC12320247; doi:10.7150/ijbs.110133)
Supplement: Supplementary file 1 — Supplementary figures. [file ijbsv21p4428s1.pdf]

Supplementary information for original paper

Fibroblast growth factor 19 disrupts cartilage development via FGFR4/β-catenin axis

Hao Chen[1], Yujia Cui[1], Jiazhou Li[1], Mengmeng Duan[1], Caixia Pi[1], Xuedong Zhou[1*], Jing Xie[1*]

[1]. State Key Laboratory of Oral Diseases & National Center for Stomatology & National Clinical Research Center for Oral Diseases, West China Hospital of Stomatology, Sichuan University, Chengdu 610041, Sichuan, China.

Supplementary Figures

Figure S1

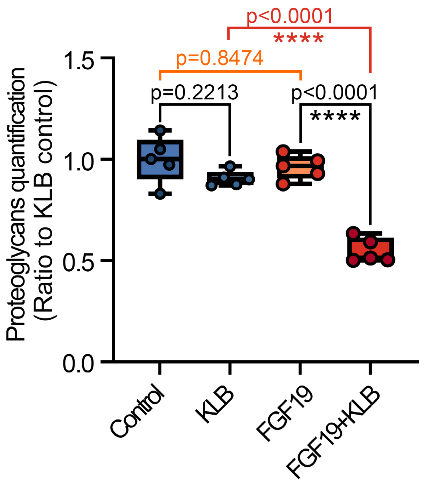

Figure S1. Quantification of the accumulation of proteoglycans in the metatarsal growth plate. Quantification was performed to confirm the changes in the accumulation of proteoglycans shown in **Figure 1e**. The significance data presented were based on two-tailed Student's t tests. The results were obtained on five regions from three independent experiments.

Figure S2

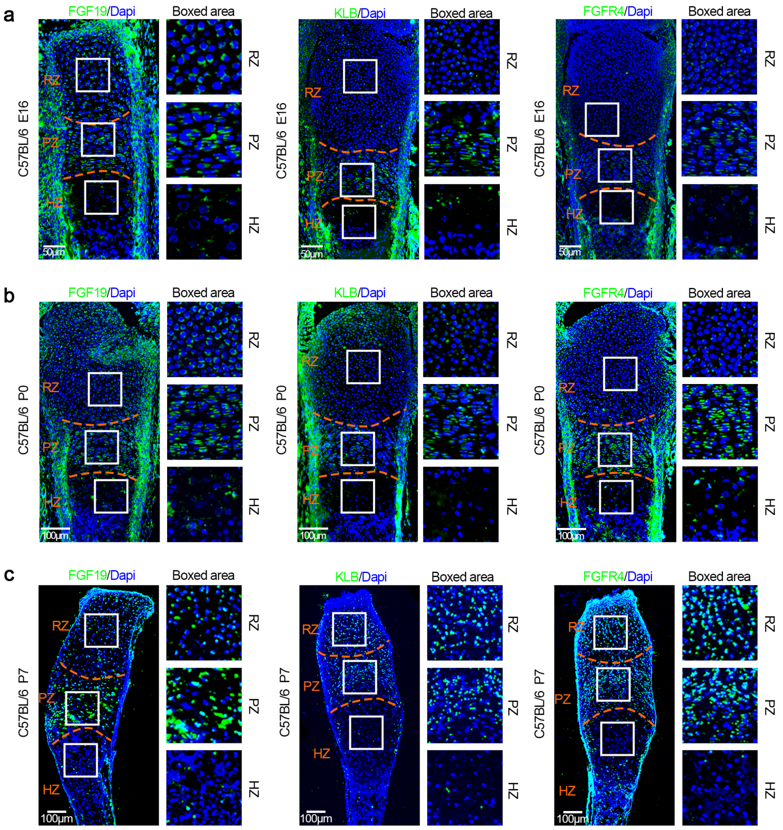

Figure S2. Spatiotemporal expression patterns of FGF19, FGFR4, and KLB in developing metatarsal growth plates. **a.** Immunofluorescence staining showing the endogenous expression of FGF19, KLB, and FGFR4 in the cartilage of the metatarsal at embryonic day 16 (E16). **b.** Immunofluorescence staining showing the endogenous expression of FGF19, KLB, and FGFR4 in the cartilage of the metatarsal at postnatal day 0 (P0). **c.** Immunofluorescence staining showing the endogenous expression of FGF19, KLB, and FGFR4 in the cartilage of the metatarsal at postnatal day 7 (P7). RZ, Resting zone, PZ, Proliferating zone, and HZ, Hypertrophic zone.

Figure S3

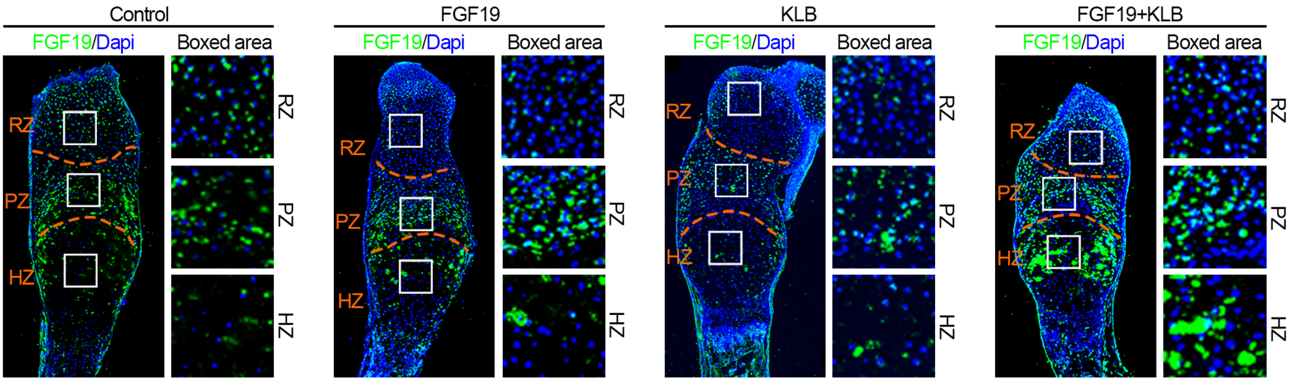

Figure S3. Immunofluorescence staining showing the changes in FGF19 in the cartilage of the metatarsal bone head induced by FGF19 and KLB for 7 days. RZ, Resting zone, PZ, Proliferating zone, and HZ, Hypertrophic zone.

Figure S4

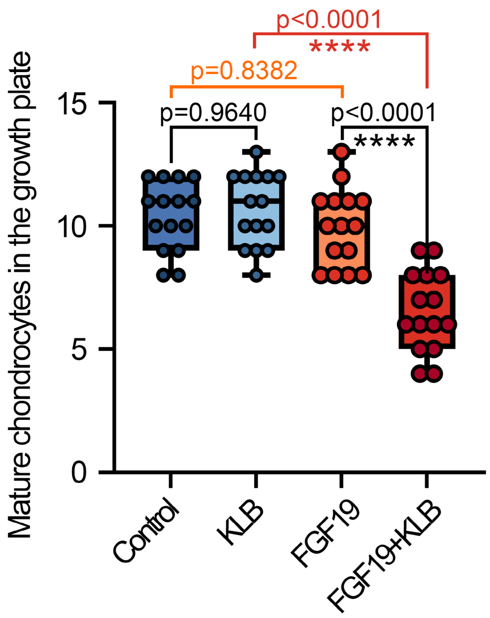

Figure S4. Quantification about the length of the cluster-like mature chondrocytes in the growth plate. Quantification was performed to show the changes in the number of cluster-like mature chondrocytes in the growth plate region. The significance data presented were based on two-tailed Student's t tests. The data were based on at least fifteen clusters of cells from three independent experiments.

Figure S5

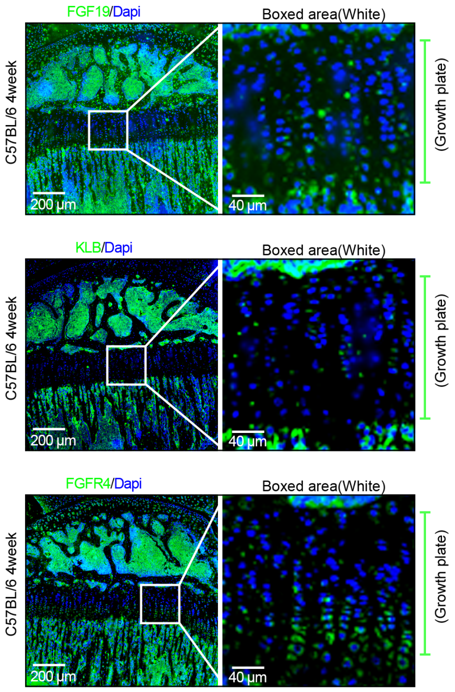

Figure S5. Immunofluorescence staining showing the endogenous expression of FGF19, KLB, and FGFR4 in articular cartilage at 4 weeks.

Figure S6

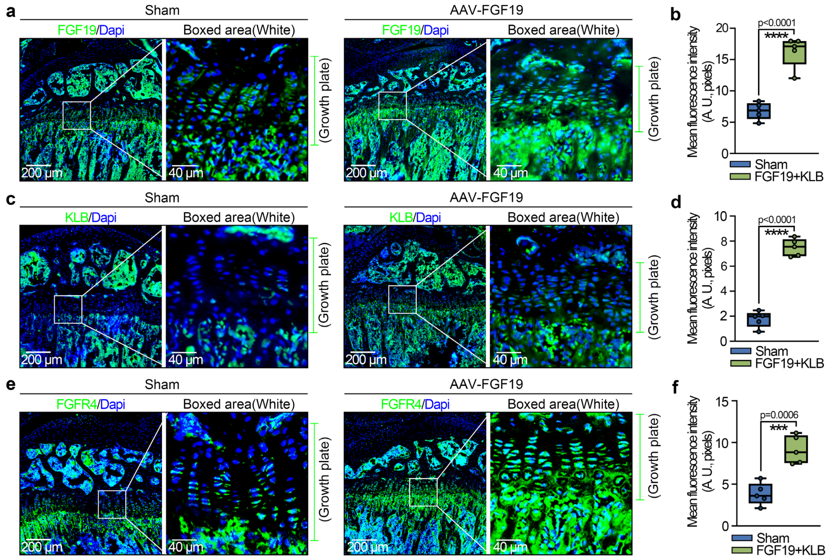

Figure S6. FGF19 overexpression *in vivo* induces increased expression of FGF19, KLB, and FGFR4. **a.** Immunofluorescence staining showing the changes in FGF19 in the growth plate in AAV-FGF19-overexpressing articular cartilage at 8 weeks. **b.** Quantitative analysis of changes in FGF19 expression in the growth plate in AAV-FGF19-overexpressing articular cartilage. **c.** Immunofluorescence staining showing the changes in KLB in the

growth plate in AAV-FGF19-overexpressing articular cartilage at 8 weeks. **d.** Quantitative analysis of changes in KLB expression in the growth plate in AAV-FGF19-overexpressing articular cartilage. **e.** Immunofluorescence staining showing the changes in FGFR4 in the growth plate in AAV-FGF19-overexpressing articular cartilage at 8 weeks. **f.** Quantitative analysis of changes in FGFR4 expression in the growth plate in AAV-FGF19-overexpressing articular cartilage.

Figure S7

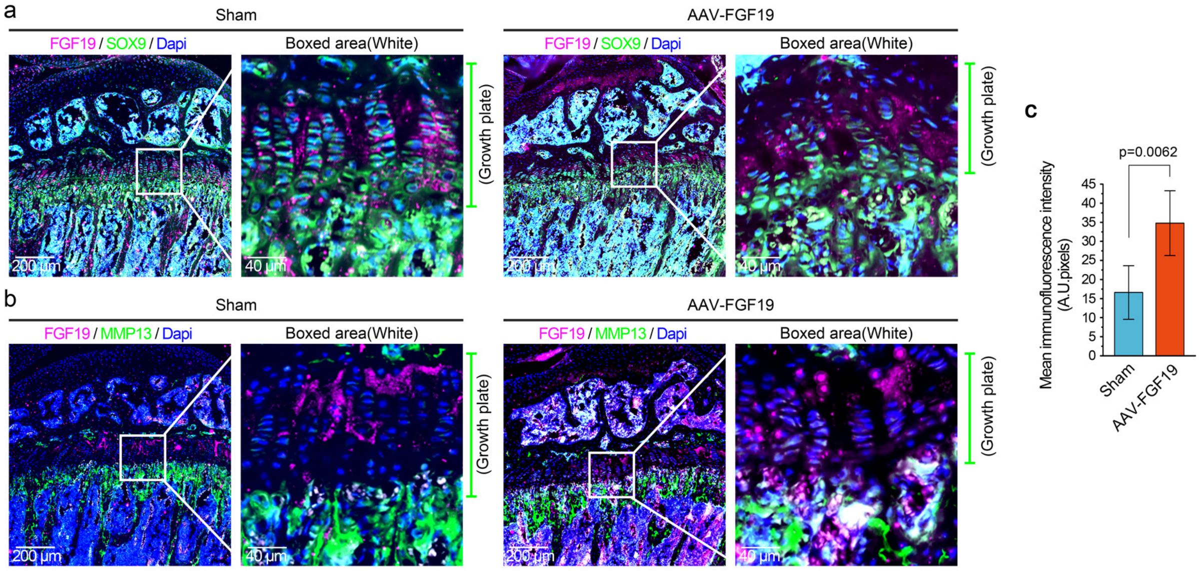

Figure S7. a. Immunofluorescence staining showing the changes in co-staining of FGF19 and SOX9 in the growth plate in AAV-FGF19-overexpressing articular cartilage at 8 weeks. **b.** Immunofluorescence staining showing the changes in co-staining of FGF19 and MMP13 in the growth plate in AAV-FGF19-overexpressing articular cartilage at 8 weeks. **c.** Quantitative analysis of changes in FGF19 expression in metatarsal growth plates after AAV-FGF19-overexpression.

Figure S8

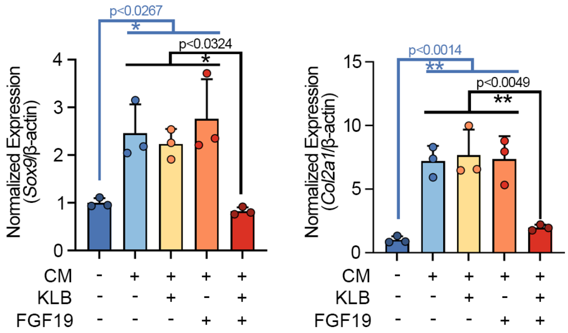

Figure S8. Representative q-PCR results showing the *Col2a1* and *Sox9* levels in BMSCs stimulated with FGF19 (200 ng/ml) in the presence of KLB (200 ng/ml).

Figure S9

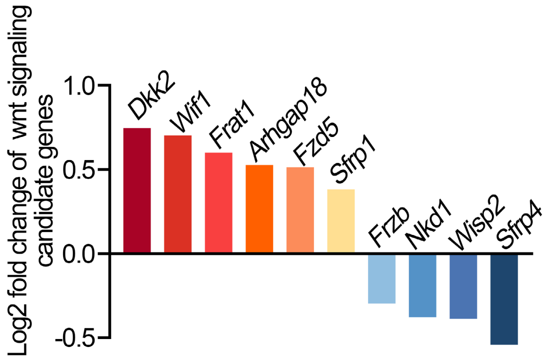

Figure S9. Ratio of wnt signaling candidate genes in chondrocytes induced by FGF19 (ratio to the KLB control group). Histogram showing the ratio of 10 wnt signaling candidate genes, i.e, *Dkk2, Wif1, Frat1, Arhgap18, Fzd5, Sfrp1, Frzb, Nkd1, Wisp2* and *Sfrp4*.

Figure S10

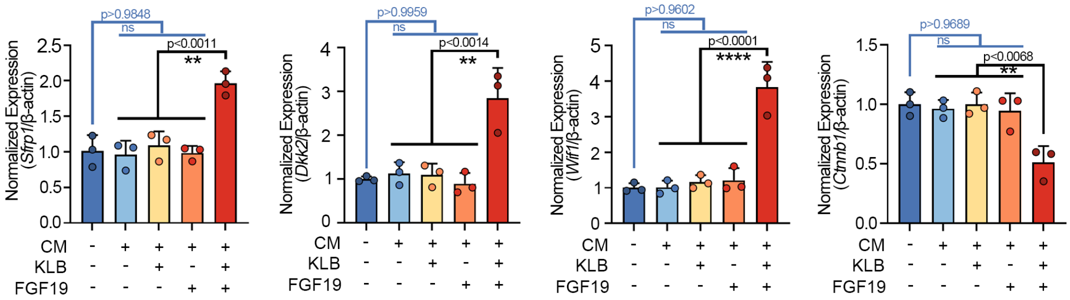

Figure S10. Q-PCR results showing that FGF19 (200 ng/ml) increased the levels of *Sfrp1*, *Wif1*, and *Dkk2* and decreased the level of *Ctnnb1* in BMSCs in the presence of KLB (200 ng/ml).

Figure S11

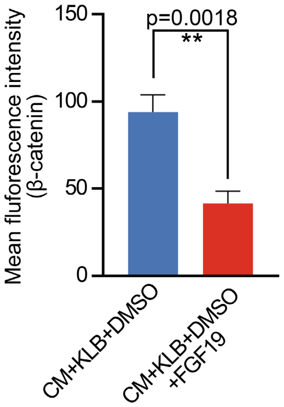

Figure S11. Quantification of mean fluorescence of intracellular β-catenin in chondrogenic BMSCs. Quantification was performed to confirm the changes in mean fluorescence of intracellular β-catenin shown in **Figure 6f**. The significance data presented were based on two-tailed Student's t tests. The result was obtained from three independent experiments.

Figure S12

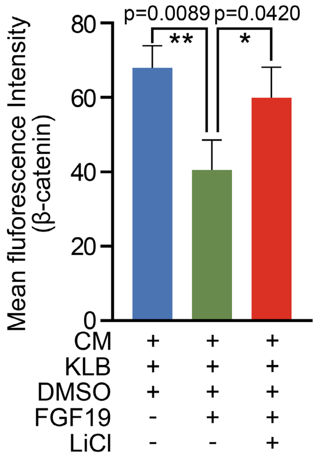

Figure S12. Quantification of mean fluorescence of intracellular β-catenin in chondrogenic BMSCs. Histograms showing the quantitative analysis of mean fluorescence of intracellular β-catenin in **Figure 7c**. The significance data presented were based on two-tailed Student's t tests. The result was obtained from three independent experiments.

Figure S13

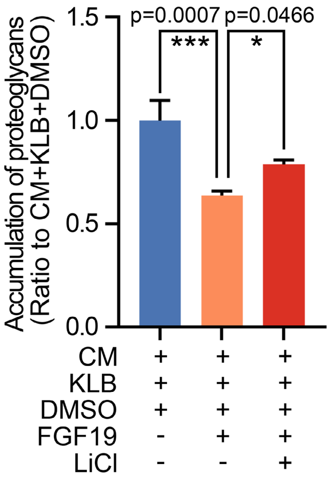

Figure S13. Quantification of the accumulation of proteoglycans. Quantification was performed to confirm that LiCl could restore the accumulation of proteoglycans that was reduced by FGF19 shown in **Figure 7e**. The significance data presented were based on two-tailed Student's t tests. The result was obtained from three independent experiments.

Figure S14

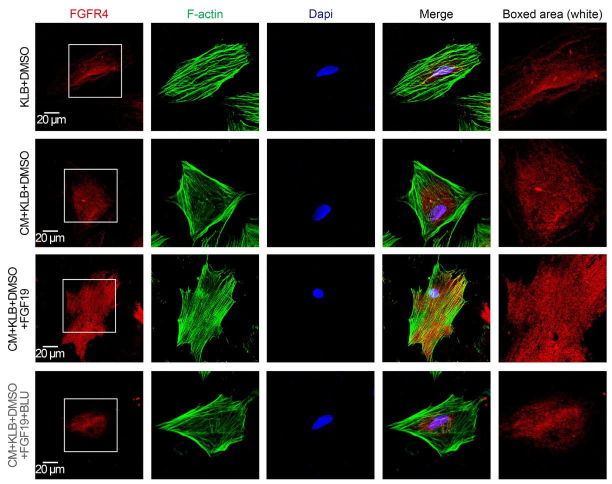

Figure S14. FGF19 induces increased expression of FGFR4 in chondrogenic BMSCs. Representative IF staining by CLSM showing the increased expression of FGFR4 in chondrocytes treated with 200 ng/ml FGF19 in the presence of KLB (200 ng/ml). Cytoskeleton, green; FGFR4, red; nucleus, blue.

Figure S15

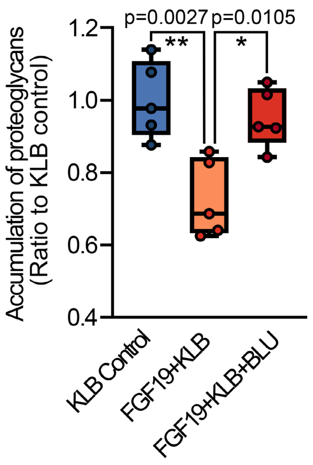

Figure S15. Quantification of the accumulation of proteoglycans in the metatarsal growth plate. Quantification was performed to confirm that BLU9931 could restore the accumulation of proteoglycans that was reduced by FGF19 shown in **Figure 8f**. The significance data presented were based on two-tailed Student's t tests. The results were obtained on five regions from three independent experiments.

Figure S16

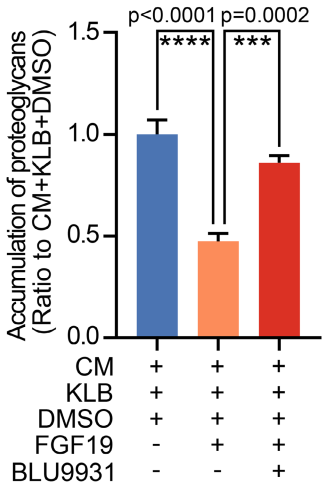

Figure S16. Quantification of the accumulation of proteoglycans. Quantification was performed to confirm that BLU9931 could restore the accumulation of proteoglycans that was reduced by FGF19 shown in **Figure 9g**. The significance data presented were based on two-tailed Student's t tests. The result was obtained from three independent experiments.

Figure S17

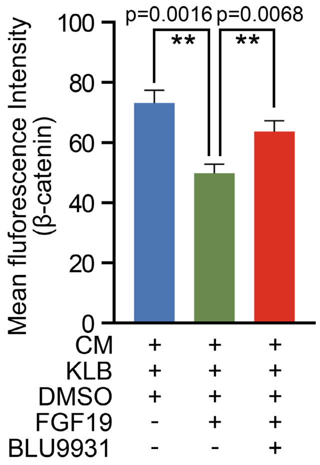

Figure S17. Quantification of mean fluorescence of intracellular β-catenin in chondrogenic BMSCs. Histograms showing the quantitative analysis of mean fluorescence of intracellular β-catenin in **Figure 9j**. The significance data presented were based on two-tailed Student's t tests. The result was obtained from three independent experiments.
